# Plateaus, rebounds and the effects of individual behaviours in epidemics

**DOI:** 10.1038/s41598-021-97077-x

**Published:** 2021-09-15

**Authors:** Henri Berestycki, Benoît Desjardins, Bruno Heintz, Jean-Marc Oury

**Affiliations:** 1 École des Hautes Études en Sciences Sociales and CNRS, CAMS, Paris, France; 2 Institute for Advanced Study, Hong Kong University of Science and Technology, Clear Water Bay, Hong Kong; 3 Université Paris-Saclay, ENS Paris-Saclay, CNRS, Centre Borelli, Gif-sur-Yvette, France; 4 Geobiomics, 75 Av. des Champs-Elysées, 75008 Paris, France

**Keywords:** Applied mathematics, Ecological epidemiology

## Abstract

Plateaus and rebounds of various epidemiological indicators are widely reported in Covid-19 pandemics studies but have not been explained so far. Here, we address this problem and explain the appearance of these patterns. We start with an empirical study of an original dataset obtained from highly precise measurements of SARS-CoV-2 concentration in wastewater over nine months in several treatment plants around the Thau lagoon in France. Among various features, we observe that the concentration displays plateaus at different dates in various locations but at the same level. In order to understand these facts, we introduce a new mathematical model that takes into account the heterogeneity and the natural variability of individual behaviours. Our model shows that the distribution of risky behaviours appears as the key ingredient for understanding the observed temporal patterns of epidemics.

## Introduction

The onset of plateaus for various indicators of the current outbreak of Covid-19 such as incidence rate or hospitalisations appears to be a rather general feature of its dynamics, along with periods of exponential growth or decay, rebounds etc. Nonetheless, there are few theoretical explanations offered to understand this phenomenon and such plateaus hardly agree with the classical SIR paradigm of epidemics.

We show here that plateaus emerge *intrinsically* in the unfolding of an epidemic. That is, plateaus arise naturally if we take into account two elements: an underlying heterogeneity and a random variability of behaviours in the population. These features are of course more realistic than assuming that the population is perfectly homogeneous with an unwavering behaviour.

To shed light on this mechanism, we propose in this paper a new mathematical model. It takes the form of a system of reaction-diffusion equations, where one variable represents the behaviour of individuals (see “Methods” section). It is natural to consider that individuals may change their behaviour from one day to the next one. We assume here that individuals’ behaviours move randomly according to Brownian motion among these classes. We show here that such a system that includes heterogeneity and variability of behaviours exhibits a richness of dynamics and in particular gives rise to intrinsic formation of plateaus, shoulders and rebounds.

Up to now, there are two main alternative explanations for the onset of plateaus. The first one is political^1^. By managing the epidemic and keeping the exponential growth at bay, without destroying the economy, a plateau appears as some kind of optimal compromise under constraint. Another approach appears in a very recent work of Weitz et al.^2^. It argues that plateaus are caused by change of behaviours due to awareness of fatalities and fatigue of the public facing regulatory mobility restrictions. In recent works, Arthur et al.^3^ and Radicchi et al.^4^ proposed models in the same spirit. More detailed discussion of related literature is provided below.

We then introduce and discuss the mathematical model. We illustrate the types of dynamics that this model gives rise to by numerical simulations. These shed light on the key role of behavioural variability to obtain plateaus, shoulders and rebounds. There, we also provide a simulation to describe the effect of introducing a second variant of the virus that yields a higher secondary epidemic peak.

To discuss the validity of our approach, we rely on observations stemming from a series of measurements, carried weekly over an extended period in the Thau lagoon area in South of France. These strikingly reveal the formation of plateaus, in some cases after a ”shoulder” pattern. These data do not include the effect of variants or of rebounds.

Next, we report on the calibration of our model on the data of the Thau lagoon. It yields a remarkable fit. We further discuss our model in more detail in the light of the measurements in the “Discussion” section below. We also show that this model also generates rebounds.

### A brief review of literature

Numerous papers^5–8^ describe the number of daily social contacts as a key variable in the spread of infectious diseases like Covid-19 insofar as it is closely related to the transmission rate. Daily social contacts are usually described in terms of age, gender, income, type of job, household size^9^, etc. Parameters that are particularly relevant in the context of viral outbreaks are also studied^10^ such as cumulative duration of such contacts, social distance, indoor/outdoor environment, etc. The very fine grain microscopic models^11^ aim at identifying such parameters in the most precise way possible. A very recent study by Di Domenico et al.^12^ takes up the data of hospitalisations in France for the past six months. Again, these exhibit striking epidemic plateaus since the beginning of 2021. The authors of this paper provide a microscopic insight of the propagation, emphasising the role of two different strains of the virus and the role of public health measures such as the curfew, school closing etc. These approaches are different from ours, as the point of view we adopt here can be seen as “mesoscopic”.

Several earlier works have considered SIR-type systems (Susceptible-Infectious-Removed) with heterogeneity. In particular, Arino et al.^13^, and, more recently, Dolbeault and Turinici^14,15^, Magal et al.^16^ have studied models with a finite number of different coefficients $\beta$. These systems are characterised by a discrete set of classes and do not involve variability. Almeida et al.^17^ considered the case of continuous classes associated with a multidimensional trait *x* to mathematically study the influence of variability of infectious individuals on the final size of an epidemic. Note that they include a diffusion term of the infectious population while we consider social diffusion of the susceptible.

Weitz et al.^2^ recently developed an SEIR-type (Susceptible-Exposed-Infectious-Removed) compartmental model with variable transmission rate coefficient, in which two main competing psychological reactions to the epidemic generate plateaus. Namely, it involves awareness of fatalities and fatigue of the public facing mobility restrictions. In this interesting model, fatigue modulates the transmission rate $\beta$ in the SEIR system by the number of cumulative deaths (and in another version the daily number of deaths). Note that this value itself is an outcome of the model and thus this model is a kind of fixed point formulation. The authors show that their model yields plateaus, “shoulder” like patterns and oscillations for the dynamics of infectious individuals.

This paper of Weitz et al. assumes a homogeneous population: at a given time, all individuals have the same transmission rate. Thus our model is quite different from theirs. The common behaviour simply changes in time by reacting to the outcome of the epidemic and this change reflects in the evolution of the transmission rate. Moreover, we note that the model is calibrated with reported death of various U.S. states and does not involve wastewater concentration measurements. Thus, even though in a different manner from ours, this work also stresses the role of variability for the observed dynamics.

DiMarco et al.^18^ have recently proposed a model close in spirit to ours but more complex. Based on kinetic theory, it describes the heterogeneity of individuals in terms of a variable $x\ge 0$ corresponding to the number of daily social contacts. Their model consists of a system of three SIR equations coupled with Boltzmann or Fokker-Planck type equations. The authors emphasise a collision type term in the resulting Boltzmann equations. This term represents changes of behaviours (that is of the values of *x*) when two individuals meet. Consequently, the present work is quite different from theirs. As a closure of their model, DiMarco et al.^18^ formally derive a so called S-SIR model. There, the variability of behaviours involves an explicit dependence of the transmission rates upon the current number of infectious individuals^19^. Because of this feature, as a matter of fact, when applied to real data, it is eventually very similar to the approach in Weitz et al.^2^.

## Results

### The Thau lagoon data

The measurement campaign concerned four wastewater treatment plants (WWTP) in the Thau lagoon area in France, serving the cities of Sète, Pradel-Marseillan, Frontignan and Mèze. The measurements were obtained by using digital PCR^20^ (dPCR) to estimate the concentration of SARS-CoV-2 virus in samples taken weekly from 2020-05-12 to 2021-01-12. We provide further details about the measurement method in the “Methods” section.

**Figure 1:**
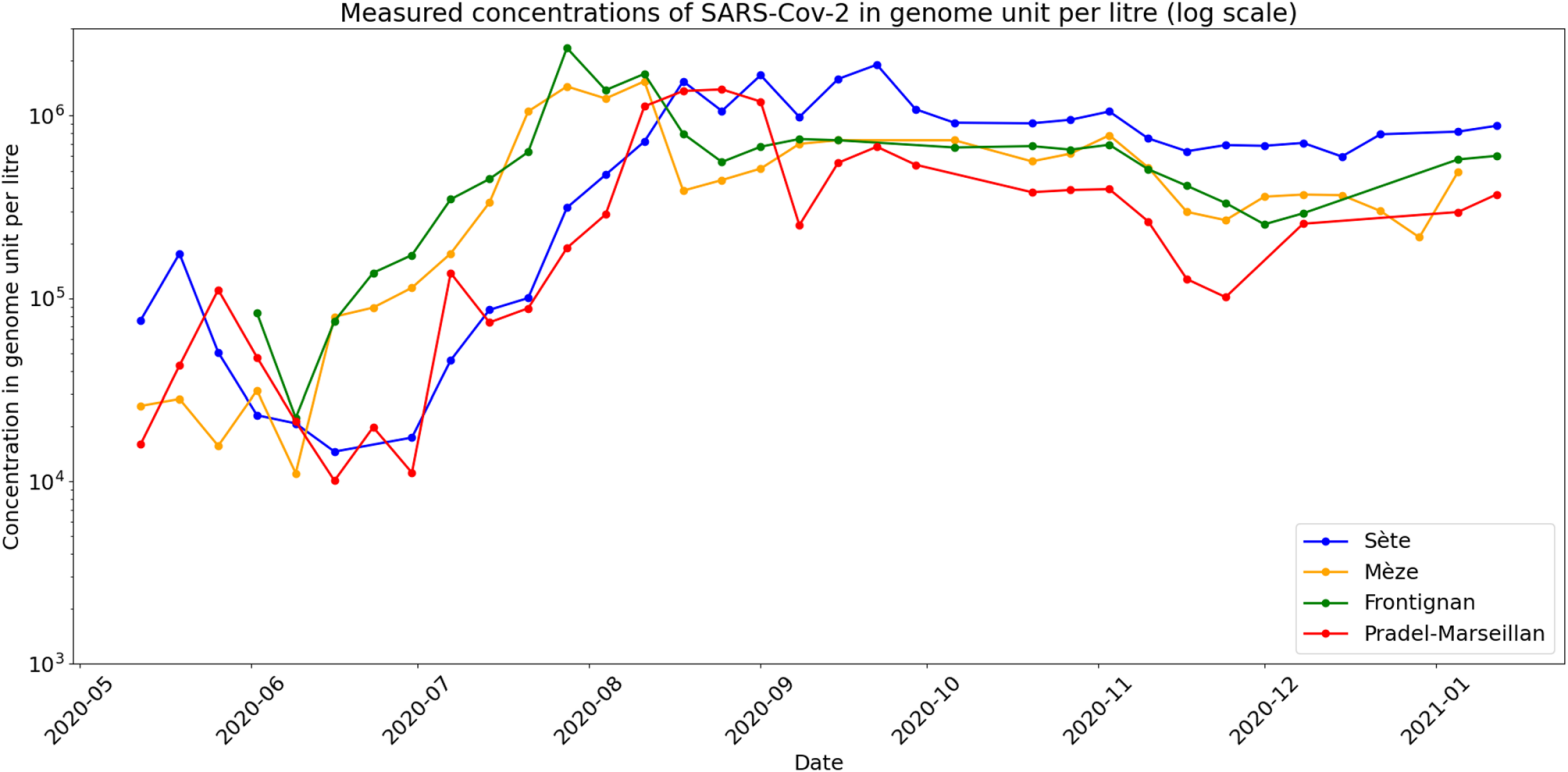
Concentrations of SARS-CoV-2 (genome units per litre in logarithmic scale) from four WWTPs in Thau lagoon, measured weekly with dPCR technology from May 12th 2020 to January 12th, 2021. Note that there are some missing points.

Figure 1 shows the outcomes in a logarithmic scale over a nine months period. We summarise now their main features.

1. An exponential phase starts simultaneously in Mèze and Frontignan WWTPs in early June.
2. The genome units concentration curves in these two places reach, again simultaneously, a plateau. It has stayed essentially stable or slightly decreasing since then.
3. The evolution at Sète and Pradel-Marseillan remarkably followed the previous two zones in a parallel way, with a two weeks lag. The measurements at Sète and Pradel-Marseillan continued to grow linearly (recall that this is in log scale, thus exponentially in linear scale), while the Mèze and Frontignan figures have stabilised ; then, after two weeks, they too stabilised at a plateau with roughly the same value as for the other two towns.
4. The measurements seem to show a tendency to increase over the very last period.

### The epidemiological model with heterogeneity and natural variability of population behaviour

The appearance of such plateaus and shoulders need not be explained either by psychological reactions or by public health policy effects. Indeed, the regulations were roughly constant during the measurement campaign and awareness or fatigue effects do not seem to have altered the dynamics over this long period of time. Witness to this is the fact that two groups of towns saw the same evolution, but two weeks apart one from the other. To understand this phenomena we propose a new model.

Given the complexity and multiplicity of behavioural factors favouring the spread of the epidemic, we assume that the transmission rate involves a normalised variable $a \in (0,1)$ that defines an aggregated indicator of risky behaviour within the susceptible population. Thus, we represent the *heterogeneity* of individual behaviours with this variable. We take *a* as an implicit parameter that we do not seek to calculate. The classical SIR model is macroscopic and the type of model we discuss here can be viewed as intermediate between macroscopic and microscopic.

The initial distribution of susceptible individuals $S_0(a)$ in the framework of a SIR-type compartmental description of the epidemic can be reasonably taken as a decreasing function of *a*. We take the infection transmission rate $a \mapsto \beta (a)$ to be an increasing function of *a*. In the Supplementary Information (SI) Appendix, the reader will find a more general version of this model involving a probability kernel of transition from one state to another. The model here can be derived as a limiting case of that more general version.

Likewise, the behaviour of individuals usually changes from one day to another^21^. Many factors are at work in this *variability*: social imitation, public health campaigns, opportunities, outings, the normal variations of activity (e.g. work from home certain days and use of public transportation and work in office on others) etc. Therefore, the second key feature of our model is *variability* of such behaviours: variations of the population density for a given *a* do not only come from individuals becoming infected and leaving that compartment but also results from individuals moving from one state *a* to another^21^. In the simplest version of the model, variability is introduced as a diffusion term in the dynamics of susceptible individuals.

#### The model

We denote by *S*(*t*, *a*) the density of individuals at time *t* associated with risk parameter *a*, by *I*(*t*) the total number of infected, and by *R*(*t*) the number of removed individuals. We are then led to the following system:

1 $$\begin{aligned} \frac{{\partial S(t,a)}}{{\partial t}} & = d{\mkern 1mu} \frac{{\partial ^{2} S(t,a)}}{{\partial a^{2} }} - \beta (a)S(t,a)\frac{{I(t)}}{N} \\ \frac{{{\text{d}}I(t)}}{{{\text{d}}t}} & = \frac{{I(t)}}{N}{\mkern 1mu} \int\limits_{0}^{1} \beta (a)S(t,a)\;da - \gamma I(t), \\ \frac{{{\text{d}}R(t)}}{{{\text{d}}t}} = & \gamma I(t), \\ \end{aligned}$$

where $\gamma$ denotes the inverse of typical duration (in days) of the disease and *d* a positive diffusion coefficient. System (1) is supplemented with initial conditions

2 $$\begin{aligned} S(0,a) = S_0(a), \quad I(0) = I_0, \quad \hbox {and} \quad R(0) = 0, \end{aligned}$$

and with zero flux condition in *a* at $a=0, 1$. In the “Methods” section below, we discuss the relation of this model with other current works.

#### A more general model

In a more general version of our model, we can consider the population of infected as also structured by the parameter *a*. The equations are as before but now we keep track of the class *a* in the infected population. The mechanism here is that a susceptible individual from class *a* can be infected by infectious from any class *I*(*t*, *b*) but then gives rise to an individual *I*(*t*, *a*) of the same parent class. We also assume that there is a diffusion of the infected behaviours. We denote by ${\mathfrak {B}}(a,b)$ the transmission rate of *S*(*t*, *a*) by *I*(*t*, *b*). For simplicity and because it is natural, we will assume that it is of the form

$$\begin{aligned} {\mathfrak {B}}(a,b)= \beta (a) \beta (b) \end{aligned}$$

where $\beta$ is as before. For full generality, we can also envision multi-dimensional parameters $a\in {\mathbb {R}}^d$, with $a_i\in (0,1)$. We are then led to the system:

3 $$\begin{aligned} \frac{{\partial S(t,a)}}{{\partial t}} & = d\;\Delta _{a} S(t,a) - S(t,a)\frac{{\beta (a)}}{N}\int\limits_{0}^{1} \beta (b)I(t,b)\;db \\ \frac{{\partial I(t,a)}}{{\partial t}} & = d^{\prime}\Delta _{a} I(t,a) + S(t,a)\frac{{\beta (a)}}{N}\int\limits_{0}^{1} \beta (b)I(t,b)db - \gamma I(t,a), \\ \frac{{{\text{d}}R(t)}}{{{\text{d}}t}} & = \gamma \int\limits_{0}^{1} I (t,b){\mkern 1mu} db, \\ \end{aligned}$$

In the SI we write further, more general, forms of this model, with ${\mathfrak {B}}(a,b)$ and more general diffusion of behaviours, that can include jumps or non-local variations. The type of models we discuss here may also shed light on the initial phase of the epidemic. We plan to investigate these questions in future work.

### Patterns generated by the model

In the next section, we will discuss how the model fits the data observed in the Thau lagoon measurements. But before that, we start by showing that the above model (1) can generate the different patterns we mentioned. For this we rely on numerical simulations without fitting real data. And indeed we obtain plateaus, shoulders, and oscillations. The latter can be interpreted as epidemic rebounds.

**Figure 2:**
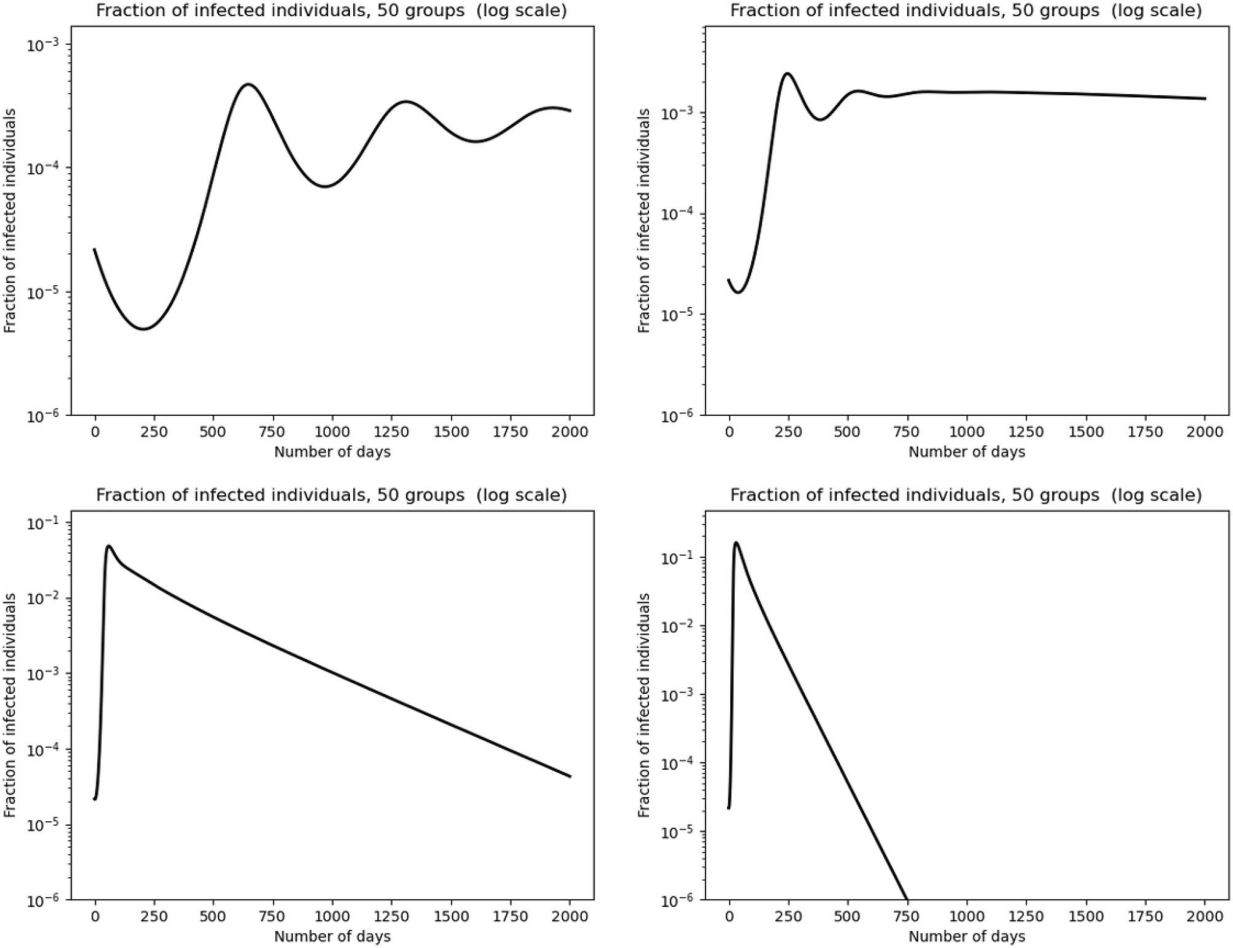
Model behaviour depending on diffusion parameter values: infected rate dynamics in logarithmic scale. From left to right and then top to bottom, graphs are associated with $d=10^{-5}$, $d=5\times 10^{-5}$, $d=10^{-3}$ and $d=5\times 10^{-3}$ (in $day^{-1}$ unit).

The key parameter here is the diffusion coefficient *d*, which controls the amplitude of behavioural variability (see Fig. 2). Large values of *d* rapidly yield homogenised behaviours, leading to classical SIR-like dynamics of infectious individuals. For very small values of *d*, the system also has a simple dynamics, in the sense that *I*(*t*) has a unique maximum, and therefore has no rebounds. We derive this in the limit $d=0$ for which we show in the SI that there are neither plateaus nor rebounds.

For some intermediate range of the parameter *d*, plateaus may appear after an exponential growth, like in the initial phase of the SIR model. A small amplitude oscillation, called “shoulder”, precedes a temporary stabilisation on a plateau, followed by a large time convergence to zero of infectious population. We also show that for small enough *d*, time oscillations of the infectious population curve, i.e. epidemic rebounds, may be generated by Model (1). Such oscillations also appear after a plateau, in a similar way to what one can see in observations.

Simulations in Fig. 2 illustrate the various patterns obtained on the dynamics of infected population as a function of the diffusion parameter. For small enough *d*, in the top left graph of Fig. 2, one can see oscillations of the fraction of infectious individuals. These oscillations cannot be achieved in the classical SIR model. In fact, the two lower graphs of that figure, for somewhat larger values of *d*, exhibit the SIR model outcomes. Indeed, for sufficiently large *d*, the system becomes rapidly homogeneous (i.e. constant with respect to *a*). Yet, such oscillations are standard in the dynamics of actual epidemics, like the current Covid-19 pandemic. The intermediate value of *d*, represented in the upper right corner of Fig. 2 shows the typical onset of a plateau at a rather high value of *I*. Note that this plateau is preceded by a first small dip and then a characteristic “shoulder-like” oscillation.

Secondary epidemic peaks are of lower amplitude than the first one, as shown in the top graphs of Fig. 2. This empirical observation leads us to conjecture that, at least in many cases, it is a general property of this model (with $\beta$ independent of time). This property would then reflect a kind of dissipative nature of Model (1). It is natural to surmise that a change of behaviours in time may generate oscillations with higher secondary peaks. Such changes result for instance from lifting social distancing measures or from fatigue effects in the population.

**Figure 3:**
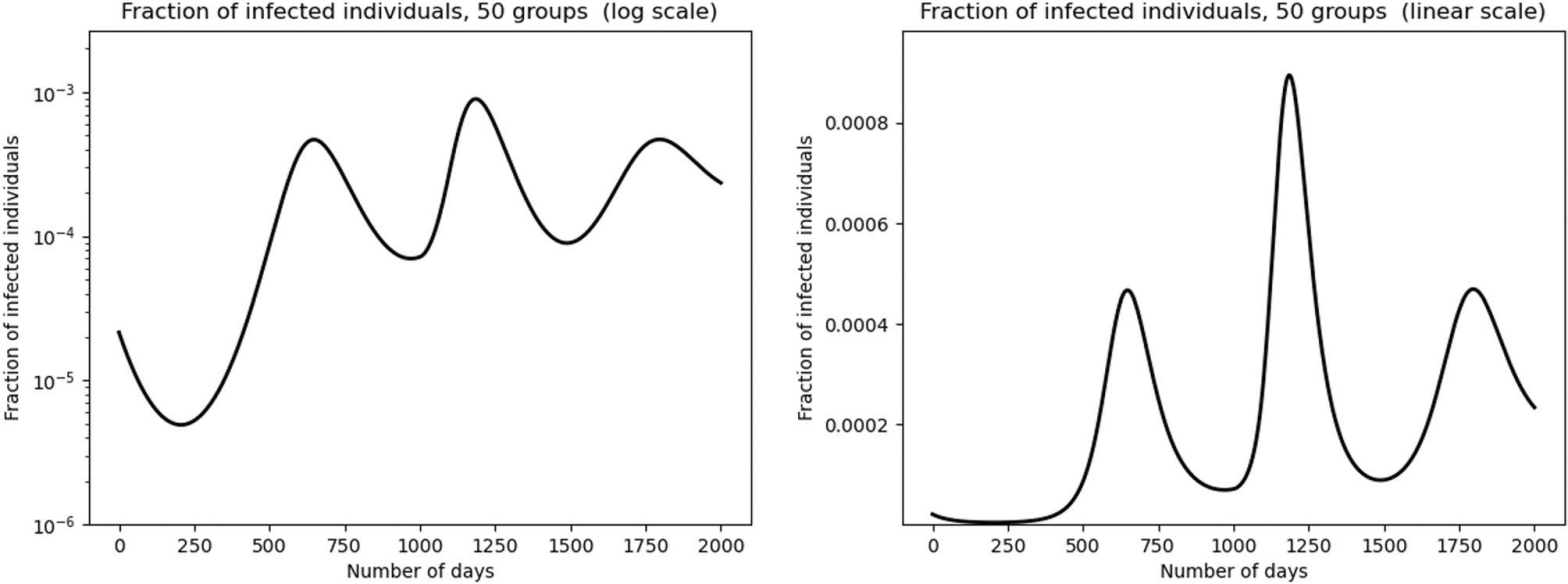
Multiple epidemic rebounds: susceptible individuals are divided into 50 discrete groups in the case where relaxation of social distancing measures starts on Day $t=1000$ and ends up on Day $t=1100$. The fraction of infected individuals in the population is represented in the left graph in logarithmic scale and in linear scale in the right graph.

We illustrate this with numerical simulations in Fig. 3. We assume a collective time modulation of the $\beta (a)$ transmission profile. That is, we replace $\beta (a)$ by $\beta (a)\varphi (t)$ for some time dependent function $\varphi$, the other parameters are the same as in the simulations shown in Fig. 2. We look at the effect of a “lockdown exit” type effect. Then, $\varphi (t)$ takes two constant values, 1 from $t=0$ to $t={1000}$ and 1.2 after $t={1100}$. In between, that is, for $t\in ({1000}, {1100})$, $\varphi (t)$ changes linearly from the value 1 to 1.2.

One can clearly see a secondary peak with higher amplitude than the first one. Note that after this peak, a third one occurs, with a lower amplitude than the second one. This third peak happens in the regime when $\beta$ is again constant in time.

#### The effect of variants

Another important factor that yields secondary peaks with higher amplitudes is the appearance of variants. Consider the situation with two variants. We denote by $I_1(t)$ and $I_2(t)$ the corresponding infected individuals. The first variant, which we call the historical strain, is associated with $\beta _1$ and $I_1(0)$ and starts at $t=0$. The variant strain corresponds to $\beta _2$ and $I_2$ and starts at Day $t=1000$. In this situation, the system (1) is extended by the following system:

4 $$\begin{aligned} \frac{{\partial S(t,a)}}{{\partial t}} & = d{\mkern 1mu} \frac{{\partial ^{2} S(t,a)}}{{\partial a^{2} }} - \left( {\beta _{1} (a)I_{1} (t) + \beta _{2} (a)I_{2} (t)} \right)\frac{{S(t,a)}}{N}, \\ \frac{{{\text{d}}I_{2} (t)}}{{{\text{d}}t}} & = \frac{{I_{2} (t)}}{N}{\mkern 1mu} \int\limits_{0}^{1} {\beta _{2} } (a)S(t,a){\mkern 1mu} da - \gamma _{2} I_{2} (t), \\ \frac{{{\text{d}}I_{1} (t)}}{{{\text{d}}t}} & = \frac{{I_{1} (t)}}{N}{\mkern 1mu} \int\limits_{0}^{1} {\beta _{1} } (a)S(t,a){\mkern 1mu} da - \gamma _{1} I_{1} (t) \\ \frac{{{\text{d}}R(t)}}{{{\text{d}}t}} & = \gamma _{1} I_{2} (t) + \gamma _{1} I_{2} (t), \\ \end{aligned}$$

**Figure 4:**
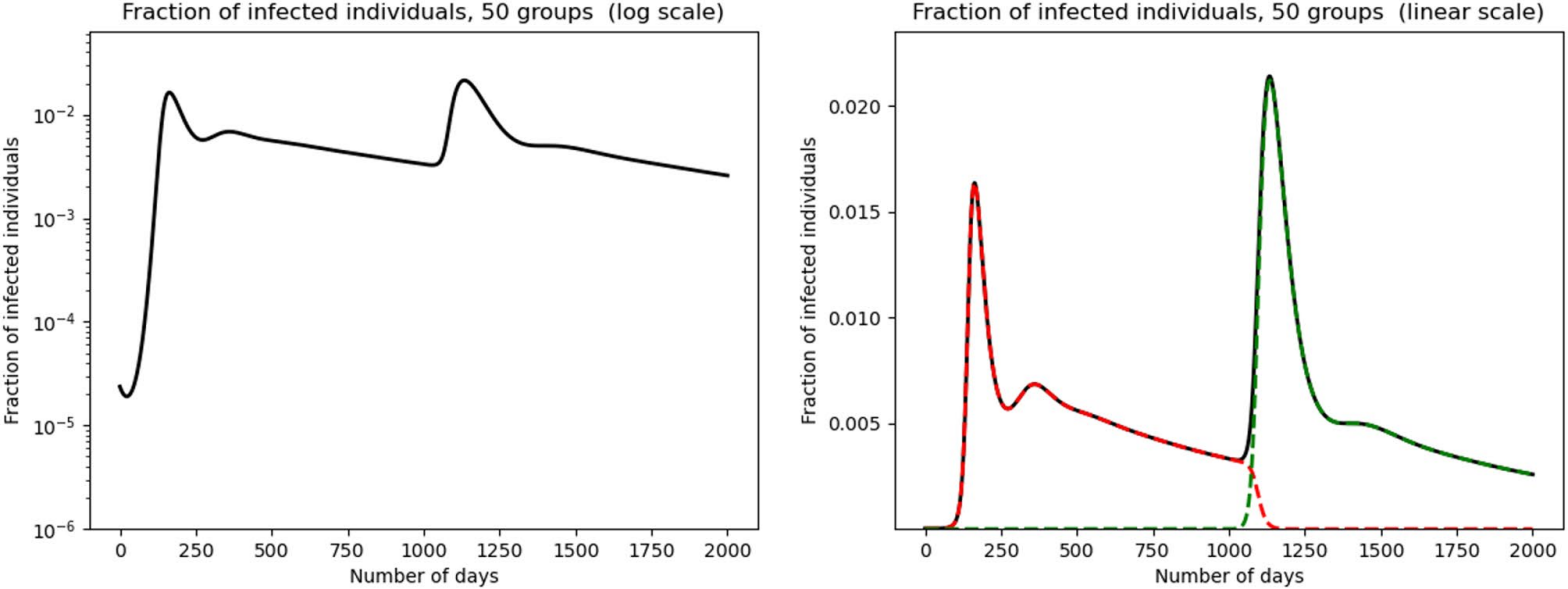
Multiple epidemic rebounds due to a variant virus: susceptible individuals are divided into 50 discrete groups in the case where a new variant appears at Day $t=1000$. The transmission rate $\beta _2$ is taken as $\beta _2(a) = 1.5 \, \beta _1(a)$, $d=0.0002$, $\gamma _1=0.1$ and $\gamma _2= 0.05$. The fraction of infected individuals in the population is represented in the left graph in logarithmic scale. The total infected population is represented in linear scale in the right graph (black curve), variant 1 in red and variant 2 in green.

The total infected population is $I(t)=I_1(t)+I_2(t)$. Figure 4 shows a simulation of this system. Before the onset of the second variant, i.e. for $t< 1000$, we observe a peak, followed by a small shoulder and a downward tilted plateau. The second variant corresponds to a higher transmission coefficient: namely, we take here $\beta _2(a) \equiv \frac{3}{2} \beta _1(a)$. When it appears at time $t=1000$, initially there is no effect, because the initial number of infectious with variant 2 is very small. Then, there is an exponential growth caused by this second variant gaining strength. The secondary peak is then higher than the first one. A very small shoulder precedes another stabilisation on a downward plateau.

Figure 4 also shows the dynamics of fractions of infected with each one of the variants. Note that the infectious with variant 1 very rapidly all but disappear at the onset of the second exponential growth phase. One might have expected that the historical strain would be gradually replaced by the new strain, merely tilting further downward the plateau. But that does not happen. Thus, it is remarkable that the historical strain gets nearly wiped out at the very beginning of the second exponential growth.

### Application to the Thau lagoon measurements

Model (1) describes the dynamics of the fraction of infectious in the population, that is $t \mapsto I(t)/N$. Therefore, we need to derive this fraction from the wastewater measurements. To this end, we use an “effective proportionality coefficient” between the two quantities. This coefficient itself is derived from the measurements (compare Section “SARS-CoV-2 concentration measurement from wastewater with digital PCR” in the “Methods” part below). Calibration of model (1) also requires fitting the values of $\gamma$, the profiles $a \mapsto \beta (a)$ and the initial distribution of susceptible individuals in terms of *a*.

**Figure 5:**
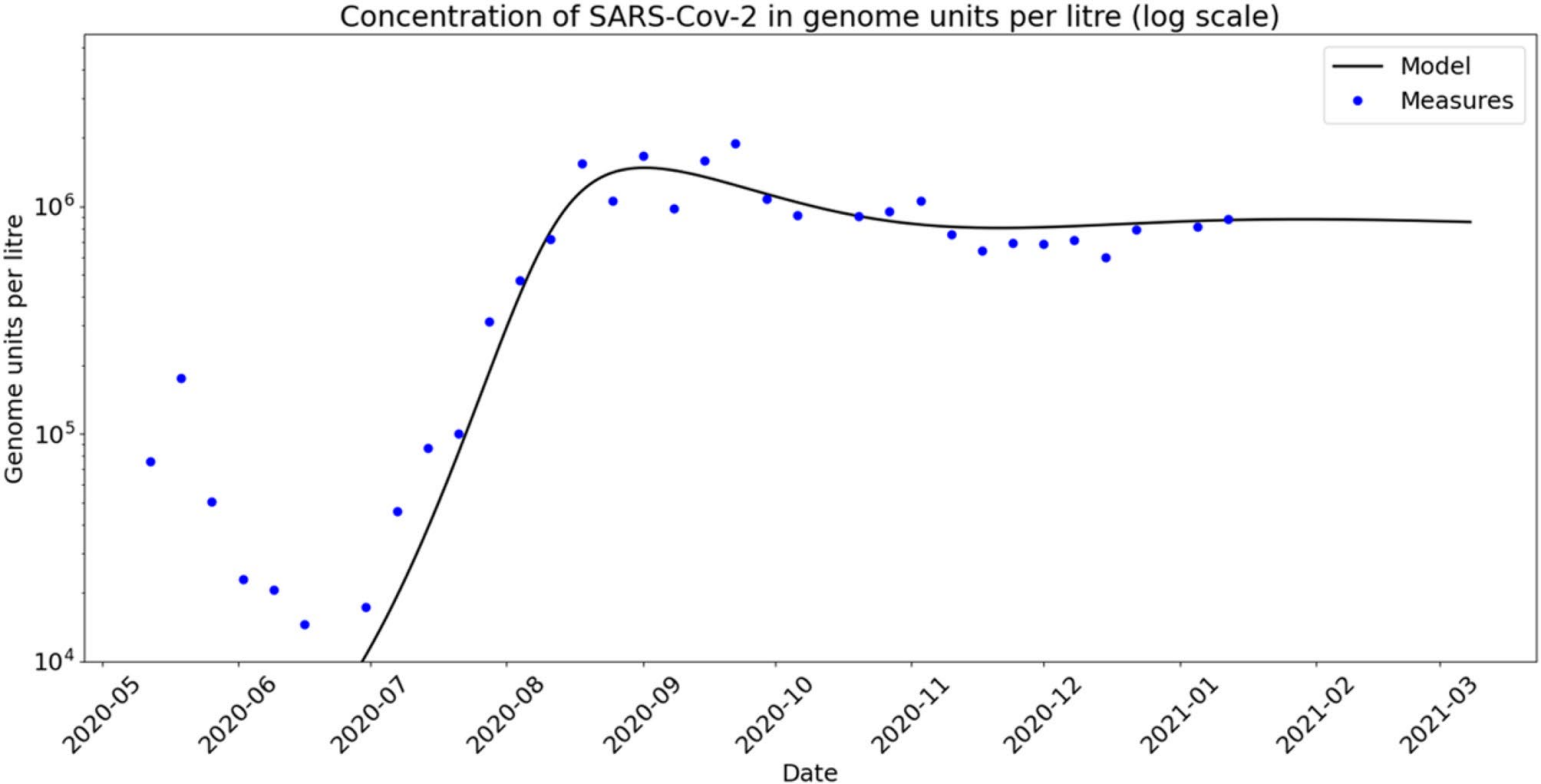
Calibrated model on Sète area: blue dots are measures of SARS-CoV-2 genome units and black curve represents the total infected individuals as an output of the model discretized into $n_g=20$ groups in *a*. Initial distribution of susceptible individuals and $\beta$ function are taken as described in supplementary information. Parameters *d* and $\gamma$ are taken as follows: $d=2.5 \times 10^{-4}$ $day^{-1}$, and $\gamma =0.1$ $day^{-1}$.

We carried this procedure and the resulting fitted curve is displayed in Fig. 5. Note that the outcome correctly captures the shoulder and plateau patterns.

**Figure 6:**
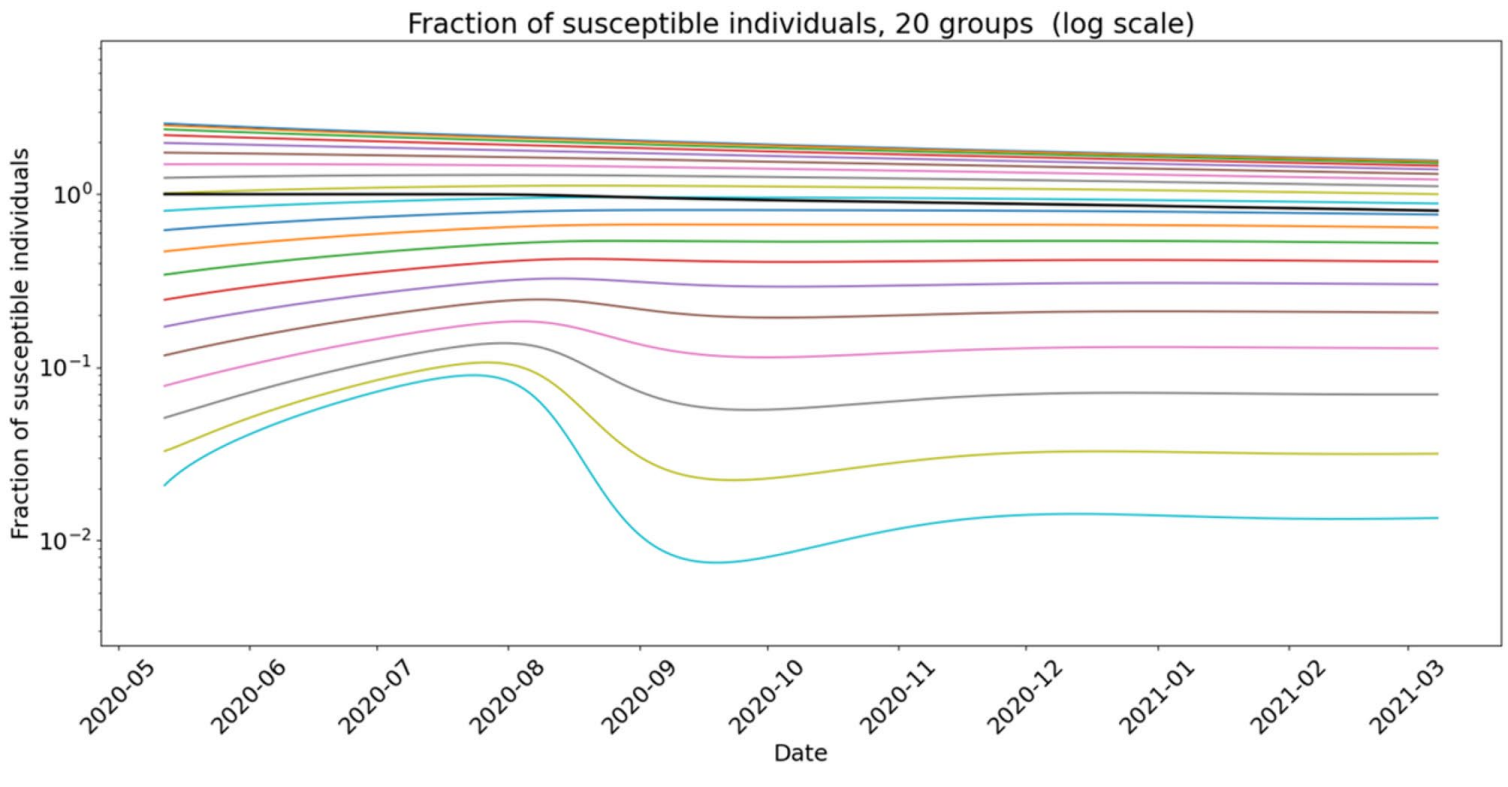
Calibrated model on Sète WWTP: density of susceptible individuals of each group for $n_g=20$. The densities of susceptible of each group is represented in colour curves as functions of time. The curves are ordered from top to bottom according to increasing *a*. The resulting average total susceptible population is represented in black. Susceptible individuals of highest *a* trait, which are represented in the bottom light blue curve exhibit a non monotonic behaviour.

The underlying dynamics of the rate of susceptible individuals is given in Fig. 6 below for $n_g=20$ groups. The lower curve illustrates the competition phenomenon between diffusion and sink term due to new infections, depending on the level of risk *a* of each state.

## Discussion

We claim that Model (1) explains the formation of plateaus and rebounds in the dynamics of the outbreak through the heterogeneity and variability of population behaviour with respect to epidemiological risk. Figure 6 shows that susceptible individuals with the riskiest behaviour, characterised by the highest $\beta$ transmission coefficient, are rapidly transferred to the infected compartment. Variability of behaviours modelled by diffusion with respect to *a* parameter then re-feeds the fringe of riskiest susceptible individuals. Parameter regimes where those two phenomena have the same order of magnitude generate patterns such as plateaus, shoulders and rebounds.

We explored above the types of patterns arising when we vary the diffusion coefficient *d*. We note that plateaus occur for an *intermediate range* of values of $d>0$, which represents the amplitude of variability. Namely, large values of *d* lead to standard SIR-type dynamics, since diffusion quickly homogenises the behaviours. When *d* decreases, plateaus starting with shoulder-like patterns appear. However, for even smaller values of *d*, oscillations arise, which can be interpreted as epidemic rebounds. From numerical experiments, the amplitude of rebounds always seems to be of smaller amplitude than the first epidemic peak. However, higher secondary peaks arise when we significantly modulate in time the transmission rate. This may represent a progressive exit from lockdown or the effect of new and more contagious variants.

The dynamics of the Covid-19 outbreak in the cities of Thau lagoon appears almost insensitive to public health regulations. In particular the second lockdown in France from 28 October to 14 December 2020 had hardly any effect. Likewise, the Christmas Holiday season also seemed to have had little influence on the observed plateaus.

**Figure 7:**
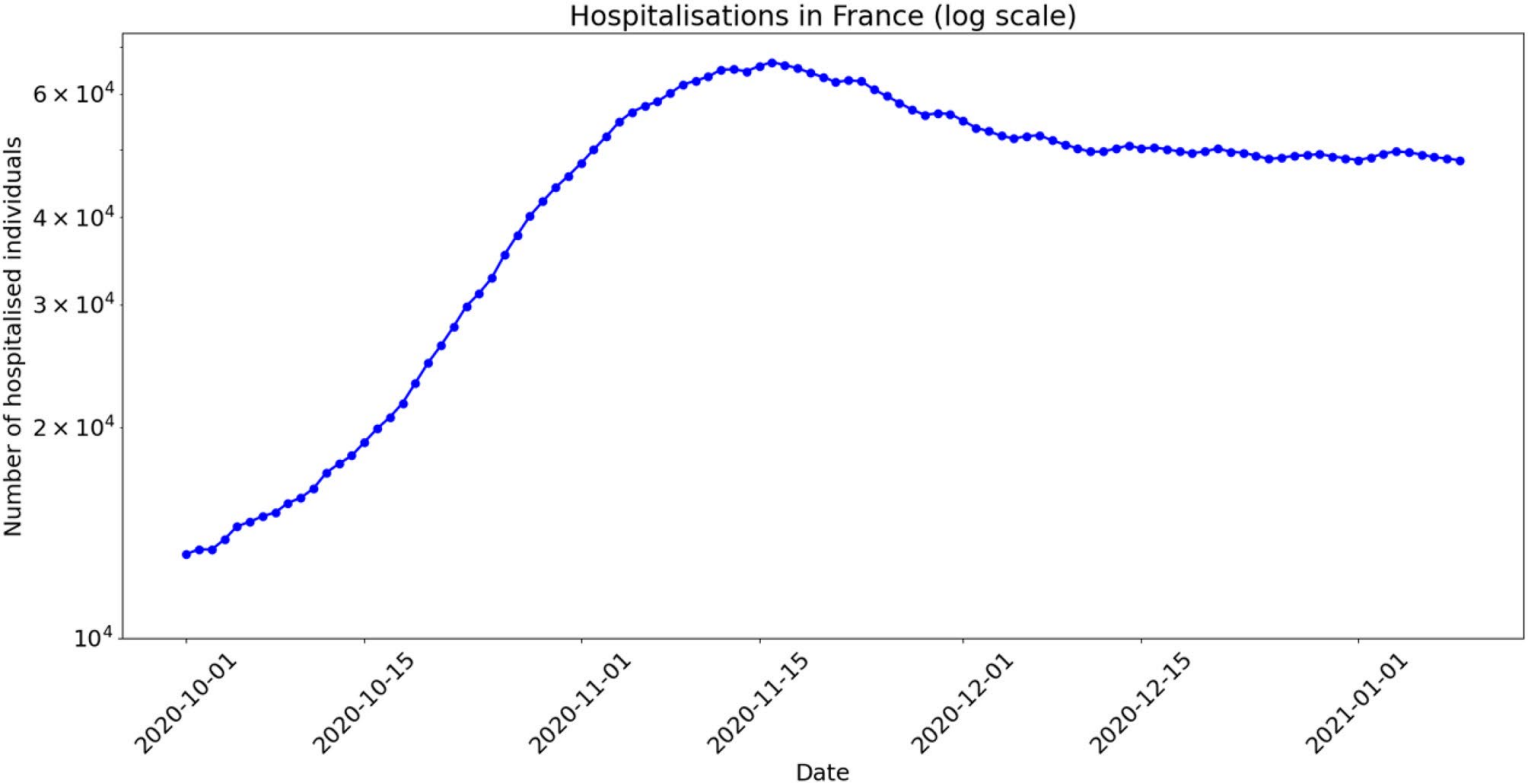
Number of hospitalised individuals in France (log scale, As from October 2020).

The number of hospitalised individuals in France over the last quarter of 2020 is represented in Fig. 7. There, the dynamics shows a growing phase followed by a shoulder and a plateau, very similar to the pattern observed in Thau lagoon.

Several noteworthy observations came out from the Thau lagoon data. First, we can see two distinct exponential growths in two separate subsets of two towns. The two graphs are parallel with a constant delay of two weeks. The first group reaches a plateau at a certain level of infected and stays thereafter essentially flat, while the second group continues to grow until it reaches about the same value of infected and then becomes essentially flat too. These remarkable observations call for interpretations. Indeed, first, they cannot simply result from public policy measures as these would have affected all these neighbouring towns in a similar fashion. Second, there likely is a spatial diffusion effect that triggered the growth in the second group coming from the first one. However, this diffusion would not explain the fact that the two groups reached the same level of plateaus.

It is worth noting that these observations are inconsistent with the classical SIR model: in this model, once an epidemic reaches a peak, it then decreases steadily by another exponential factor. In other words, a plateau requires an effective ${{\mathscr {R}}}_t$ number^22,23^ of approximately one: ${{\mathscr {R}}}_t\sim 1$. However this would be hard to sustain over such a long period of time as observed because the susceptible population is depleted. It also appears difficult to explain that this occurs exactly at the same plateau level for two distinct populations.

## Conclusions

In this paper we reported on precise concentration measurements of SARS-CoV-2 in wastewater upstream of four WWTPs around the Thau lagoon in South of France during the nine months period from May 2020 to January 2021. These observations exhibit plateau and shoulder patterns. Such characteristics, along with rebounds, are widely observed in epidemics. We provided here an explanation by considering that the population is heterogeneous in the level of risk in the individual behaviours, which random changes from day to day. Indeed, we show here that the combination of heterogeneity and variability leads to a constant replenishment of the population of susceptible individuals with higher risks, thus feeding as it were the epidemic. This mechanism explains the formation of plateaus in the dynamics of the epidemic and also accounts for oscillations and rebounds.

To substantiate this claim, we proposed a mathematical model for epidemics that explicitly involves heterogeneity and variability. The model takes the form of an SIR system with diffusion of behaviours. To get a model as parsimonious as possible^24^, we only assumed the diffusion of risks among susceptible and that a single continuous and one-dimensional risk variable *a* characterises the behaviour type.

Numerical simulations of this system indeed show exactly this type of shape that we observed, with shoulders preceding long plateaus, and rebounds. Without any change in the behaviours, the secondary peaks, although they can be important, are of a lesser amplitude than the first peaks. Here we show further that a one time change in the transmission coefficients may generate rebounds with higher amplitudes. Such changes typically occur when social distancing measures are lifted.

We also explored the effect of introducing a variant strain of the virus, with a slightly higher transmission rate. We also achieved higher amplitude rebounds in this framework. The analysis of the presence of the two strains shows that the historical strain is replaced quite abruptly by the more transmissible one, earlier than one would guess from its intrinsic evolution.

To further validate this model, we fitted it on the data collected in the Thau lagoon area. Here we did not invoke any external effects such as fatigue or change of public health measures. Indeed, over the concerned period, there were arguably few and minor changes. Furthermore, the two weeks delay between the two curves we observed precludes such effects. Indeed, two towns reached the plateau two weeks earlier than the other two, the latter continued to see an increase, until roughly the same level was reached two weeks later. The calibration of our model on the Thau lagoon observations yielded a very good agreement with the data.

### Perspectives and extensions of the model

There are several directions to extend our work. First, one can consider more general forms of the model as the one introduced in (3).

Then, one might envision a more precise approach to the parameter *a* beyond the notion of unit of contact. One can take into account e.g., duration and circumstances of contacts. It appears natural to use “risky sociability” in computing the transmission rate. One may also consider multi-dimensional versions of the parameter. Understanding quantitatively the change of behaviour as a function of this variable *a* may require an interdisciplinary avenue of research.

Likewise, it is quite natural to assume that the variability of behaviour also depends on *a* rather than being uniformly distributed. We discuss such an extension in the SI where we show that it leads to an equation with drift terms. We leave developments in this direction to further work.

Another important aspect that transpires in the Thau lagoon data, is the spatial spreading that takes place in the epidemic. In a recent paper, the first author of this study and collaborators^25^ have proposed a model at the country level with a quantitative approach to spatial diffusion in France. The study of diffusion at a smaller scale that we might call mesoscopic and its inclusion in the framework we propose here are promising perspectives.

## Methods

### The epidemiological model with heterogeneity and variability

The model we develop here extends the classical SIR compartmental approach by taking into account heterogeneity and variability of behaviours on the susceptible population. The total population is assumed to be constant equal to *N*. That is, we do not take into account incoming or outgoing populations, nor demographic changes. This is a standard assumption in the Covid-19 studies. Actually, one might want to dispense with it if one is to consider a significant amount of travellers, especially during vacation periods or because there is a lockdown that brings many people to leave large cities.

At time *t*, the population of susceptible is structured by *a* and *t* and is described by its density *S*(*t*, *a*). We assume that the total number of infected is given by *I*(*t*) and that of the removed by *R*(*t*). We do not distinguish from which population strata the infected individuals come from.

One can think of *a* as a trait parameter roughly describing the level of risk a given susceptible individual is taking with respect to Covid-19. It is normalised so that $0\le a \le 1$, $a=0$ being associated with very cautious people and $a=1$ to people with very risky behaviour. This implicit variable represents for instance the number of social contacts per day of an individual, taking into account their length, whether social distancing is observed, if it takes place indoor or outdoor, in a more or less crowded environment, or if individuals wear a mask etc. Thus, it can be seen as a lumped variable that represents a global risk score. In this context, the impact of political restrictions can easily be represented by a modification of the distribution law of the behaviours.

We observe that in this model, we adopt the point of view that the behaviour distribution affects the *risk takers* in the susceptible population rather than the behaviour of infected individuals. Indeed, it appears more natural to consider that the susceptibles face an ambient distribution *I*(*t*). For instance, the choice to go to a crowded bar where there *might be* a super-spreader, is reflected in variable *a*. Individuals often vary their behaviour, because for instance of fatigue effects for people who have heeded too strongly social distancing calls or, on the contrary, for people who have been reckless and see other people fall sick and consequently become somewhat more cautious. Thus, the potential reservoir of individuals for a given stratus level *a* is not static and it is more important than would appear at first glance.

Hence, we consider that the variations of *S*(*t*, *a*) do not only come from individuals becoming infected and leaving that compartment but also results from individuals moving from one stratus to another. Here we assume that this shuffling of behaviours follows Brownian motion. We are then led to System (1) presented in the Result section above. At the end-points of the interval (0, 1) for *a*, we impose homogeneous Neumann (zero flux assumption) boundary conditions:

5 $$\begin{aligned} \displaystyle \frac{\partial S(t,0)}{\partial a} = \frac{\partial S(t,1)}{\partial a} = 0. \end{aligned}$$

Note that in this system the total population *N* is conserved by the dynamics:

$$\begin{aligned} N(t) := \int _0^1 S(t,a) \, da + I(t) + R(t) = N(0). \end{aligned}$$

In the SI, we derive this system from more basic considerations and we describe some of its mathematical properties. There, we further discuss more general systems. In particular we consider the framework where the variability itself depends on the trait *a*.

It is enlightening to keep track of the fraction of infected coming from specific strata. To this end, we can introduce a variable *I*(*t*, *a*) representing the number of infected that came from stratus *a*. It is given by the solution of the equation:

6 $$\begin{aligned} \left\{ \begin{aligned}&\displaystyle \frac{\partial I(t,a)}{\partial t} = \beta (a) S(t,a) \frac{I(t)}{N} - \gamma I(t,a), \\&I(0,a) = I_0(a),\\ \end{aligned} \right. \end{aligned}$$

It is straightforward that this is consistent with the definition of the total population of infected, that is:

$$\begin{aligned} I(t) = \int _0^1 I(t,a)\, da. \end{aligned}$$

We may include then here the effect that infectious individuals with high *a*’s are more likely to infect other susceptible. This just amounts to replace *I*(*t*) in system (1)–(6) by

$$\begin{aligned} J(t) := \int _0^1 \beta (a) I(t,a) \, da \end{aligned}$$

in (1)–(6). We are then led to Model (3).

### SARS-CoV-2 concentration measurement from wastewater with digital PCR

The teams of the local authorities of the Thau lagoon, *Syndicat Mixte du Bassin de Thau* (or SMBT) collected samples every Tuesday from each of the four WWTPs. Each sample consisted of a compound of 24 hourly samples.

I.A.G.E. (INGENIERIE ET ANALYSE EN GENOME EDITING, 2700 route de Mende 34980 Montferrier-sur-Lez, France) developed a diagnostic method to detect very low concentrations of SARS-CoV-2 in such wastewater samples. This method combines an optimised extraction process with a DNA quantification based on a digital PCR (dPCR) targeting region of the RdRp (IP2/IP4)^20^ (This method has been submitted by IAGE to the European Patent Office the 31st of December 2020 under the application number EP20306715.2). The measures produced identical results within three significant digits between the two targets.

In contrast to classical quantitative real-time PCR (qRT-PCR), dPCR allows the *absolute* quantification of low concentration levels of target sequences of nucleic acid molecules from DNA or RNA samples. dPCR outperforms qRT-PCR with respect to accuracy^26^ and repeatability of measurements; it is also has a much lower detection threshold (about 20 times lower)^20^.

Among recently achieved wastewater measurement campaigns in sewers^27–34^, it seems that none of them exploit these measurements in a dynamical model. This is probably due to the uncertainties associated with qRT-PCR measurements and to the difficulty of translating genome unit concentrations into numbers of infected individuals. As outlined in Ahmed et al.^27^, the rate of infected individuals within the population served by the instrumented WWTP may be related to the measured genome unit concentration through a proportionality relation:

7 $$\begin{aligned} \frac{Infected\,persons}{Total\,population} = \lambda \cdot \frac{genome\,units}{litre\,wastewater} \end{aligned}$$

where

8 $$\lambda = \frac{\displaystyle {\frac{{litre\;wastewater}}{{{\text{ }}day \cdot {\text{ }}total\;population}}}}{\displaystyle {\left( {\frac{{{\text{ }}g\;faeces}}{{{\text{ }}day \cdot {\text{ }}person}}} \right) \cdot \left( {\frac{{{\text{ }}genome\;units}}{{{\text{ }}g\;faeces}}} \right)}}{\text{ }}$$

Still, the individual variability of each parameter of Eq. (8) is very high, as pointed out in several works^27,35,36^ (see also references therein). Moreover, transport of wastewater from the emission point to the WWTP involves additional significant phenomena identified in Hart et al.^37^: virus degradation over time, usually modelled by exponential decay law where the half life depends on temperature. Therefore, a bottom-up approach estimating each component of Eq. (8) from literature is not realistic because of the huge variability and uncertainties of these factors.

Instead, we develop here an original approach to estimate an *effective* value parameter $\lambda$ by viewing $\lambda$ as one of the parameters in the optimisation process in order to fit the model to the data. The reason why we are thus able to determine $\lambda$ lies in the richness of the data set combined with the complex dynamics allowed by the nonlinear model (1). Indeed, the capture of the dynamics of concentrations by the model, including the exponential growth phase, followed by the formation of shoulder-like and plateau patterns, adequately constrains the estimation of the value of this effective parameter $\lambda$. We computed this parameter for the WWTP of Sète by a least square minimisation process over the time interval from 9 June 2020 to 12 January 2021 covering the preceding three phases. Indeed, since we are interested in these phases, for the sake of clarity we chose to ignore the observations prior to this period. This procedure led us to the choice of $\lambda ^{-1} = 111,230,001$ (in genome unit per litre), a figure that seems of the correct order of magnitude.

By this data-driven optimisation procedure, we thus relate the rate of infected people to the measured concentration of SARS-CoV-2. We plan to further investigate and extend this approach in future work.

Such virus concentration time series, essentially proportional to the fraction of people infected by Covid-19, provides an accurate quantitative method to monitor the epidemic. Furthermore, as well known^29,32,34^, the appearance of the virus in wastewater precedes the observations of the disease and therefore yields a remarkable early warning system, ahead of hospital counts. Moreover, it reflects all infectious people regardless of whether they are symptomatic or asymptomatic.

## Supplementary Information

Supplementary Information.

## Data Availability

The datasets and python code used in this study are freely available from the following URL: https://www.dropbox.com/sh/wjoydsmi2lqzwnu/AAAUkBEf__BHgYu_VOw1AkIAa?dl=0.

## References

[1] Haug N (2020). Ranking the effectiveness of worldwide COVID-19 government interventions. Nat. Hum. Behav..

[2] Weitz J, Park S, Eksin C, Dushoff J (2020). Awareness-driven behavior changes can shift the shape of epidemics away from peaks and toward plateaus, shoulders, and oscillations. PNAS.

[3] Arthur R (2020). Adaptive social contact rates induce complex dynamics during epidemics. BioRxiv..

[4] Radicchi F, Bianconi G (2020). Epidemic plateau in critical susceptible-infected-removed dynamics with nontrivial initial conditions. Phys. Rev. E.

[5] Stroud P, del Valle S, Sydoriak S, Riese J, Minszewski S (2007). Spatial dynamics of pandemic influenza in a massive artificial society. J. Artif. Soc. Soc. Simul..

[6] Ibuka Y (2016). Social contacts, vaccination decisions and influenza in Japan. J. Epidemiol. Community Health.

[7] Leung K (2017). Social contact patterns relevant to the spread of respiratory infectious diseases in Hong Kong. Nat. Sci. Rep..

[8] Diekmann Odo, Heesteerbeek H, Britton T (2013). Mathematical Tools for Understanding Infectious Diseases Dynamics.

[9] Zhang J (2020). Changes in contact pattern shape the dynamics of the Covid-19 outbreak in china. Science.

[10] Béraud G (2015). The French Connection: The first large population-based contact survey in France relevant for the spread of infectious diseases. PLOS ONE..

[11] Knock E. The (2020). Sars-cov-2 epidemic in England: Key epidemiological drivers and impact of interventions. Sci. Transl. Med..

[12] Di Domenico L, Sabbatini C, Pullano G, Lévy-Bruhl D, Colizza V (2021). Impact of January 2021 curfew measures on SARS-CoV-2 B.1.1.7 circulation in France. Euro Surveill.

[13] Arino J, Davis J, Hartley D, Jordan R (2005). A multi-species epidemic model with spatial dynamics. Math. Med. Biol..

[14] Dolbeault J, Turinici G (2021). Social heterogeneity and the Covid-19 lockdown in a multi-group SEIR model. MedRxiv.

[15] Dolbeault J, Turinici G (2020). Heterogeneous social interactions and the COVID-19 lockdown outcome in a multi-group SEIR model. Math. Model. Nat. Phenom..

[16] Magal P, Seydi O, Webb G (2018). Final size of a multigroup sir epidemic model: Irreducible and non-irreducible modes of transmission. Math. Biosci..

[17] Almeida Luis (2021). Final size and convergence rate for an epidemic in heterogeneous population. Math. Models Methods Appl. Sci..

[18] Dimarco, G., Perthame, B., Toscani, G., & Zanella, M. Social contacts and the spread of infectious diseases. *arXiv: Physics and Society* (2020).

[19] Korobeinikov A, Maini P (2005). Non-linear incidence and stability of infectious disease models. Math. Med. Biol..

[20] Pasteur Institute Protocol Paris (WHO), Real-time RT-PCR assays for the detection of SARS-CoV-2. https://www.who.int/docs/default-source/coronaviruse/real-time-rt-pcr-assays-for-the-detection-of-sars-cov-2-institut-pasteur-paris.pdf (2020).

[21] Chan H, Skali A, Savage D, Stadelmann D, Torgler B (2020). Risk attitudes and human mobility during the covid-19 pandemic. Nat. Sci. Rep..

[22] Roques L, Klein E, Papaïx J, Sar A, Soubeyrand S (2020). Using early data to estimate the actual infection fatality ratio from covid-19 in france. Biology.

[23] Thieme H (2009). Spectral bound and reproduction number for infinite-dimensional population structure and time heterogeneity. SIAM J. Appl. Math..

[24] Bertozzi A (2020). The challenges of modeling and forecasting the spread of covid-19. PNAS.

[25] Roques L, Bonnefon O, Baudrot V, Soubeyrand S, Berestycki H (2020). A parsimonious approach for spatial transmission and heterogeneity in the covid-19 propagation. R. Soc. Open Sci..

[26] Suo T (2020). ddpcr: A more sensitive and accurate tool for sars-cov-2 detection in low viral load specimens. MedRxiv.

[27] Ahmed W (2020). First confirmed detection of sars-cov-2 in untreated wastewater in Australia: A proof of concept for the wastewater surveillance of covid-19 in the community. Sci. Total Environ..

[28] Haramoto E (2020). First environmental surveillance for the presence of SARS-CoV-2 RNA in wastewater and river water in Japan. Sci. Total Environ..

[29] Medema G (2020). Presence of SARS-Coronavirus-2 RNA in sewage and correlation with reported covid-19 prevalence in the early stage of the epidemic in the Netherlands. Environ. Sci. Technol. Lett..

[30] Miyani B (2020). SARS-Cov-2 in Detroit wastewater. J. Environ. Eng..

[31] Wurtzer S (2020). Evaluation of lockdown effect on SARS-CoV-2 dynamics through viral genome quantification in waste water, Greater Paris, France, 5 March to 23 April 2020. Euro Surveill.

[32] Randazzo W (2020). SARS-CoV-2 RNA titers in wastewater anticipated covid-19 occurrence in a low prevalence area. Water Res..

[33] d’Aoûst P (2021). Quantitative analysis of SARS-Cov-2 RNA from wastewater solids in communities with low covid-19 incidence and prevalence. Water Res..

[34] Peccia J (2020). Measurement of SARS-CoV-2 RNA in wastewater tracks community infection dynamics. Nat. Biotechnol..

[35] Foladori P (2020). Sars-cov-2 from faeces to wastewater treatment: What do we know? A review. Sci. Total Environ..

[36] Wölfel R (2020). Virological assessment of hospitalized patients with COVID-2019. Nature.

[37] Hart O, Halden R (2020). Computational analysis of SARS-CoV-2/COVID-19 surveillance by wastewater-based epidemiology locally and globally: Feasibility, economy, opportunities and challenges. Sci. Total Environ..

