# Supplementary material for: Plateaus, rebounds and the effects of individual behaviours in epidemics
Source: Sci Rep. 2021 Sep 15;11:18339. doi: 10.1038/s41598-021-97077-x (PMC8443568; doi:10.1038/s41598-021-97077-x)
Supplement: Supplementary file 1 — Supplementary Information. [file 41598_2021_97077_MOESM1_ESM.pdf]

Plateaus, Rebounds and the Effects of Individual Behaviours in Epidemics

Authors: Henri Berestycki, Benoît Desjardins, Bruno Heintz, Jean-Marc Oury

Supplementary information

The classical SIR model with time dependent transmission rate

The classical SIR compartmental model[1] uses a dynamics governed by mass-action laws. It assumes that individuals are homogeneously mixed and that every individual is equally likely to interact with every other individual. It reads:

$$\begin{cases} \dfrac{dS}{dt} = -\dfrac{\beta IS}{N} \\ \dfrac{dI}{dt} = \dfrac{\beta IS}{N} - \gamma I \\ \dfrac{dR}{dt} = \gamma I \end{cases} \qquad (1)$$

Single location growth and behavioural changes.

Changes of behaviour driven by phenomena such as awareness of fatalities or fatigue with respect to mobility restrictions can be modelled by a time modulation of the infection rate β.

To start with, we analyse this problem on synthetic data. Namely, we consider an exact dynamics given by the SIR model with parameter changes on two given dates. We now take part of the resulting points as given observations. The goal is to carry an optimisation procedure where we try to identify the dates and magnitudes of these changes i.e. the different values of β that come into play. Later on we will consider the Thau lagoon data.

Figure S1 represents a piece-wise constant modulation in time with reduction of social interactions down to 70% starting Day $t = 25$ followed by an increase to 50% on Day $t = 50$.

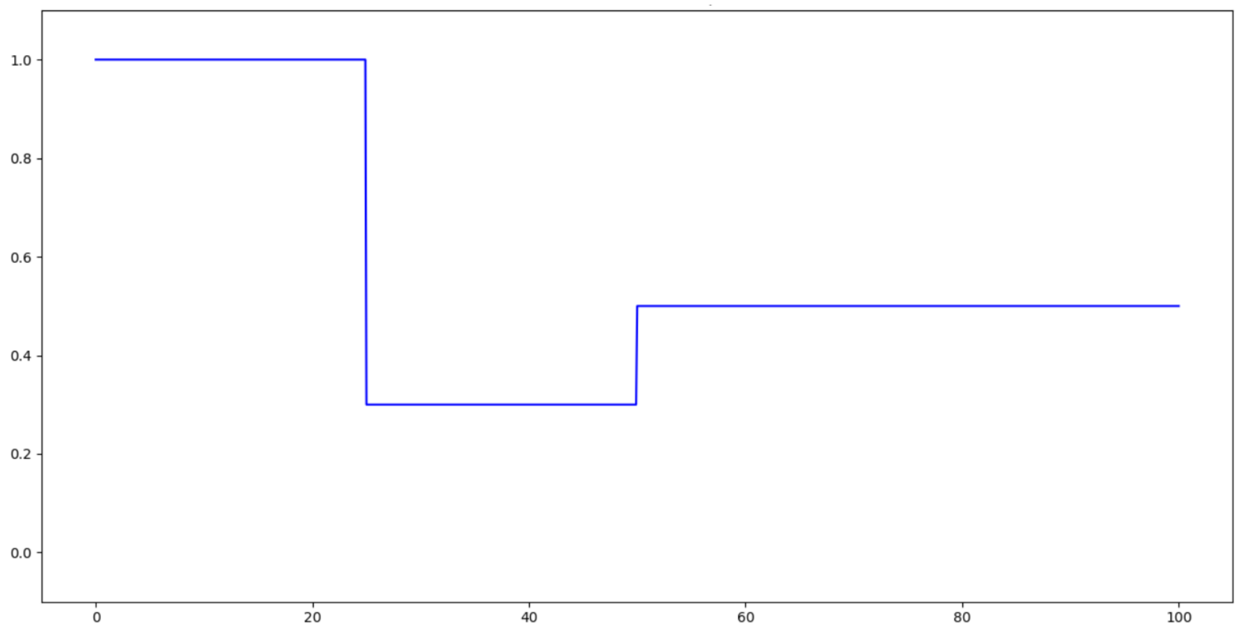

Figure S1. Time modulation function associated with collective behaviour modulation as a function of time (day numbers)

The effect on such modulation leads to the interruption of the growth phase followed by a lower decrease for later times.

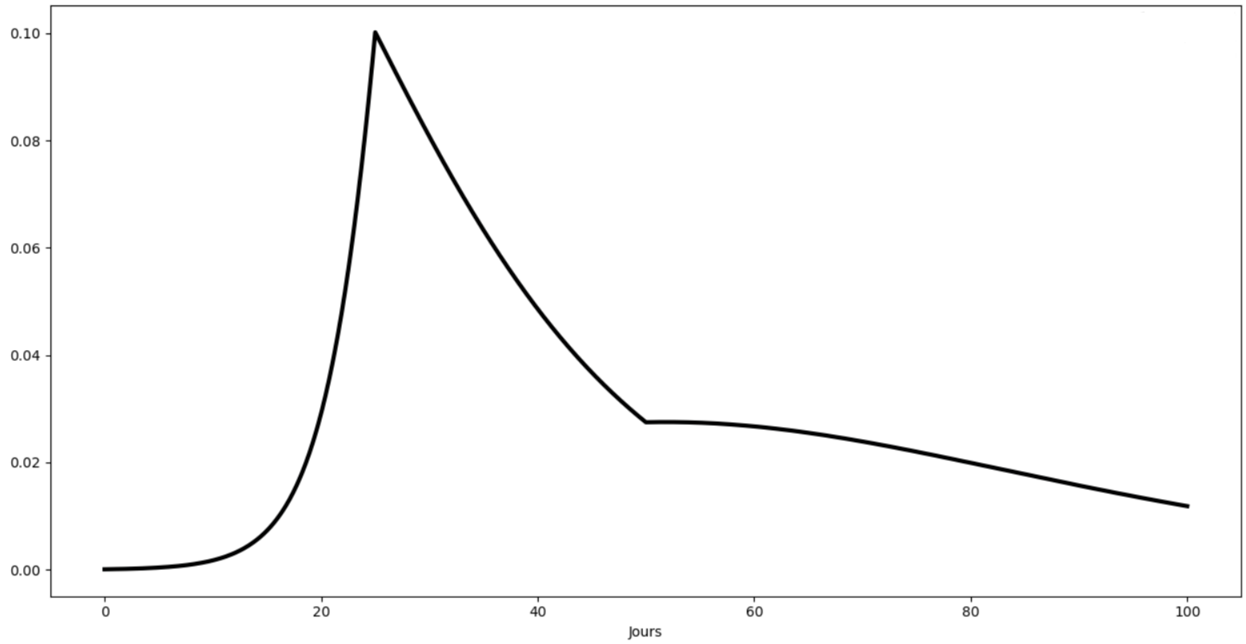

Figure S2. Rate of infected individuals as a function of days

We developed a toolbox based upon PYGMO[2] particle swarm optimisation algorithms in order to solve the type of inverse problem we encounter here. We consider a time sampled version of infection rates in the total population. We allow a given number of transition times associated with behaviour changes, in between which the coefficient β is constant. The problem then is to determine in an optimal way the SIR model parameters, initial conditions, the transition times and the values of the β's in between. Using a least square objective function, we obtained a satisfactory convergence towards the dynamics of the initial model and associated modulation function.

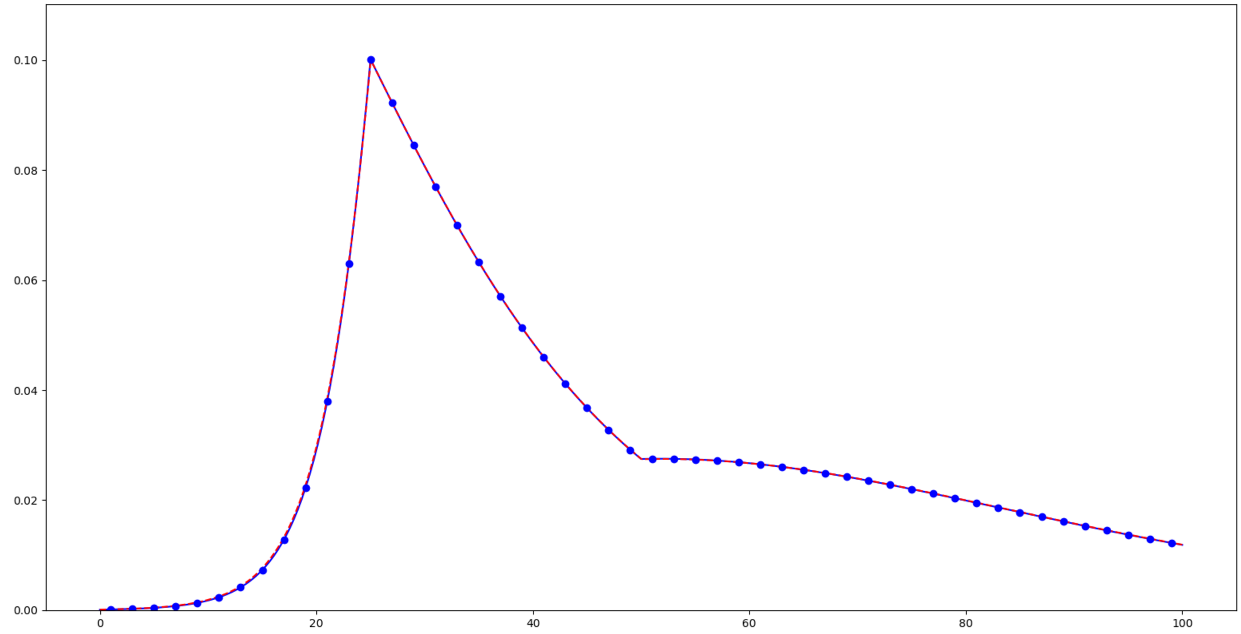

Figure S3. Rate of infected individuals as a function of days as a result of the inverse problem: blue dots correspond to daily sampling of synthetic data, red line to the resulting SIR model.

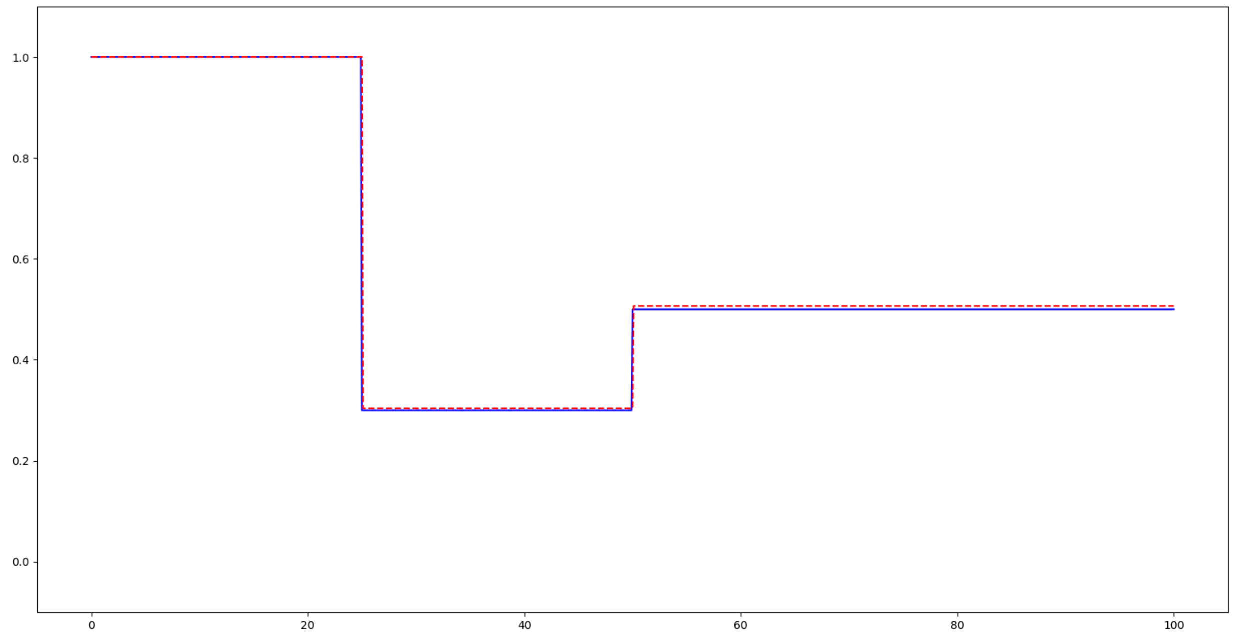

Figure S4. Modulation of collective behaviour as a result of the inverse problem

We developed a similar approach with noise added to sampled synthetic data, with satisfactory convergence both in terms of dynamics of infection rate (Figure S5) and modulation functions (levels and time changes in Figure S6).

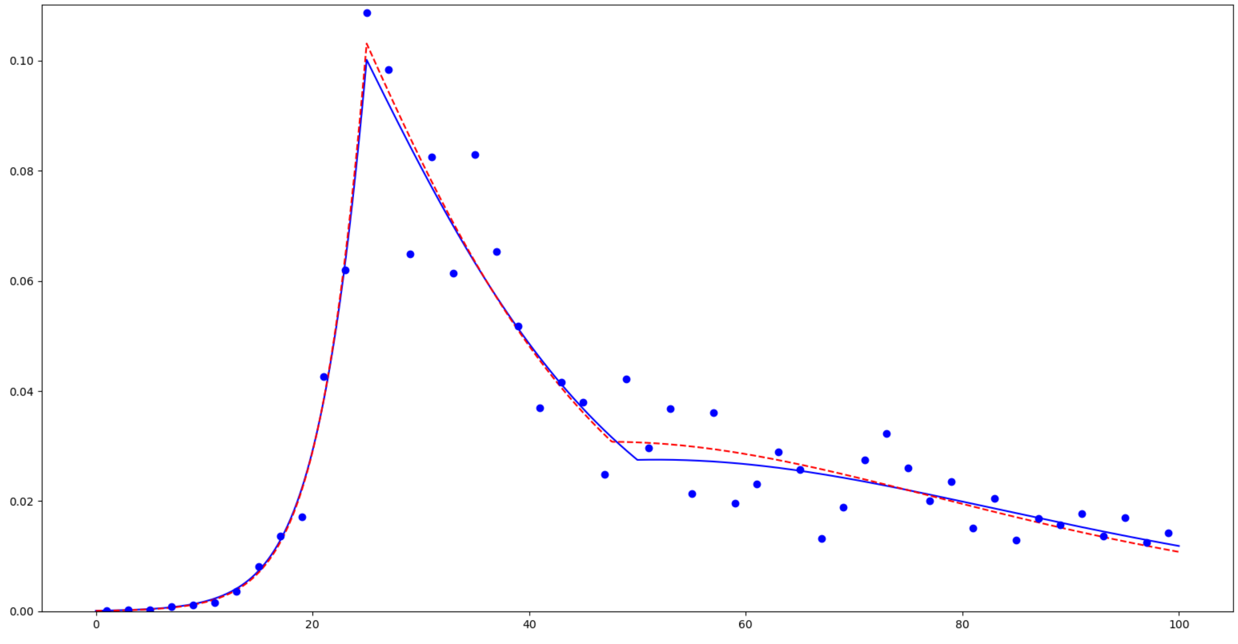

Figure S5. Inverse problem with noisy synthetic data: blue dots correspond to daily sampled synthetic data with noise, blue and red curves respectively to the initial synthetic model and to the result of the inverse problem.

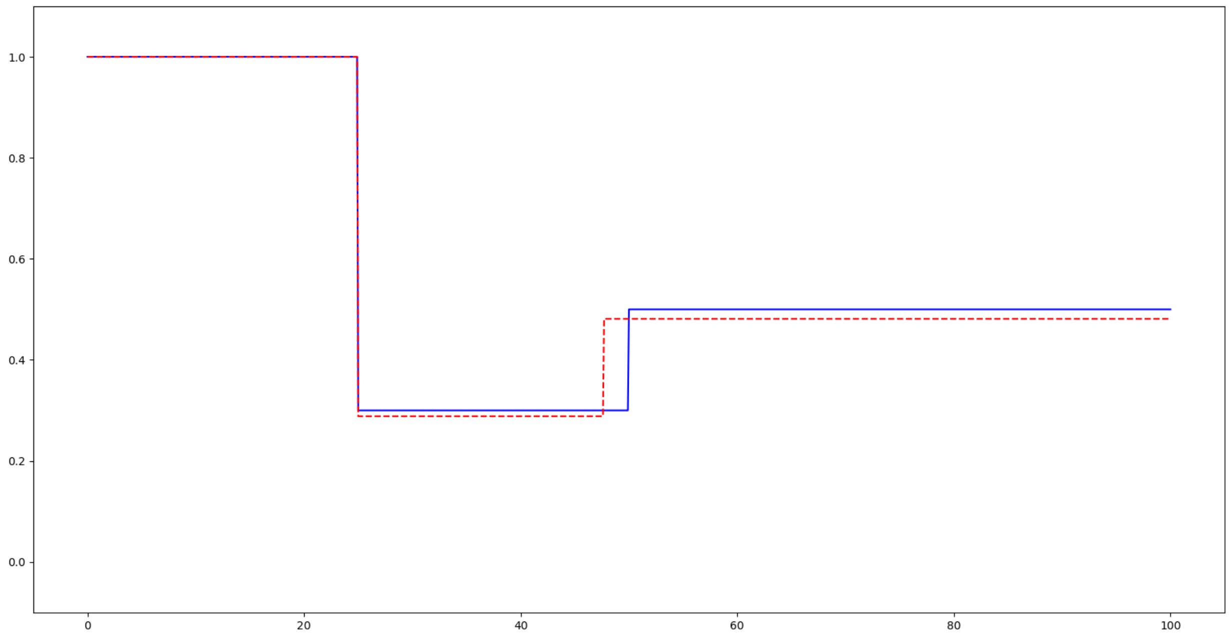

Figure S6. Original modulation function in blue and result of the inverse problem in red.

A model with change of behaviour at discrete times in Thau lagoon.
We applied the preceding algorithmic approach on real data of the four WWTP measurement campaigns of the Thau lagoon. In order to keep the model parsimonious, we used the same time modulation function of β for all the four cities together. The transition dates and levels have to be determined.

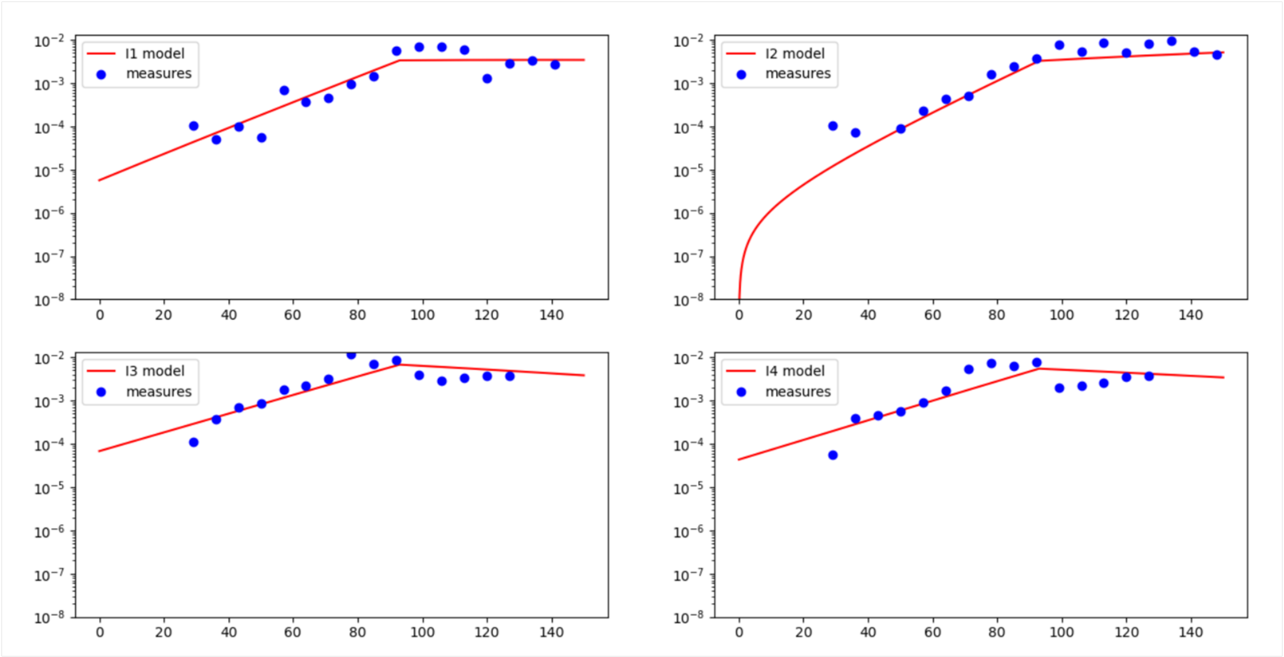

Figure S7. Change of behaviour in logarithmic scale in the Thau lagoon (initial day $t = 0$ being associated with May 12th, 2020). The red curve represents the model output (rate of infected population) and the blue dots correspond to measurements. Zones I_1, I_2, I_3 and I_4 respectively correspond to Sète, Mèze, Frontignan and Pradel-Marseillan.

The above results show that the optimal capture of the transition from exponential growth to plateau type dynamics occurs

on August 10th, 2020. The resulting piecewise constant modulation function in time is represented in Figure S8 below.

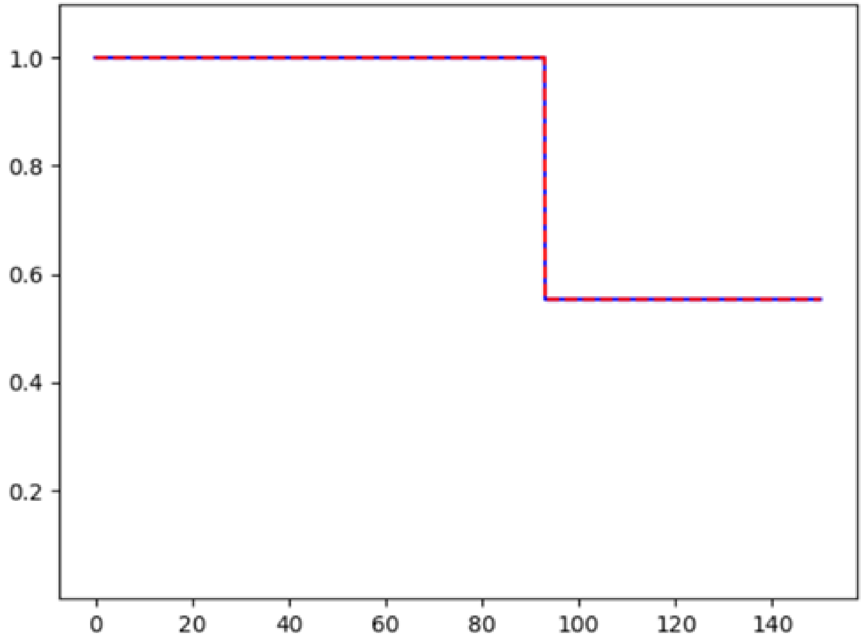

Figure S8. Time modulation of collective behaviour in Thau lagoon as a result of the inverse problem.

Even though the plateau dynamics seems to be correctly represented in Figure S7, the main shortcoming of this is that it does not really explain the observations of the Thau lagoon data. Indeed, the model does not explain why the four zones reach the same infection rate level associated with the plateau regime and why there is a two weeks delay. Furthermore, it does not yield a shoulder effect, as seen in the data.

One may use spatial diffusion to describe the delay between the zones (Frontignan, Mèze) on the one hand and (Sète, Pradel-Marseillan) on the other hand. But it also fails to explain why there are two parallel curves. We plan to come back to spatial diffusion in future work.

Derivation of the Model

This section is concerned with the formal derivation of the additional diffusion term in the dynamics of susceptible individuals. As previously emphasised, this diffusion term stems from the combination of the heterogeneity of behaviours and their variability in time. A natural case to consider here is to assume that the shuffling of behaviours happens according to Brownian motion. Namely, individual risk traits move according to the process

$$da_t = \sqrt{2}\sigma\, dW_t,$$

where $\sigma > 0$ is the possibly a-dependent diffusion, and W_t is a Brownian motion in $(0,1)$ with reflection conditions at the end-points of the interval. By the Fokker-Planck equation, in the absence of epidemic, an initial distribution of population $S(0,a)$ gives rise to $S(t,a) = \mathbb{E}[S(0,a_t)]$ governed by

$$\frac{\partial S}{\partial t} = \frac{\partial^2[\sigma^2(a)S]}{\partial a^2}. \tag{2}$$

To consider the dynamics of the epidemic, we are thus led to the following more general version:

$$\begin{cases} \dfrac{\partial S(t,a)}{\partial t} = d\,\dfrac{\partial^2[\sigma^2(a)S(t,a)]}{\partial a^2} - \beta(a)S(t,a)\dfrac{I(t)}{N}, \\ \dfrac{\mathrm{d}I(t)}{\mathrm{d}t} = \dfrac{I(t)}{N}\displaystyle\int_0^1 \beta(a)S(t,a)\,da - \gamma I(t), \\ \dfrac{\mathrm{d}R(t)}{\mathrm{d}t} = \gamma I(t). \end{cases} \tag{3}$$

The first equation involves drift and other additional terms. The numerical and theoretical analysis of the case where σ depends on a, and the corresponding extensions of the Fokker-Planck equation with drift terms, seem relevant from a modelling viewpoint.

In the simple case where $\sigma^2(a) = d$ where d is a constant, one ends up with the first equation of our system (see System (4) below).

One could also envision a multi-dimensional variable $a = (a_1, \dots, a_p)$ to encompass various behavioural characteristics that could possibly be related to sociological observations. In this case, one would be led to a higher dimensional partial differential equation. where the term $\partial^2_{aa} S$ is replaced by the Laplace operator $\Delta_a S$ as we did in the general model presented in Methods section. Such developments would be useful to improve the model and its applicability.

Simple properties of Model (4)

For the convenience of the reader, we recall the model introduced in the main paper

$$\begin{cases} \dfrac{\partial S(t,a)}{\partial t} = d\,\dfrac{\partial^2 S(t,a)}{\partial a^2} - \beta(a) S(t,a) \dfrac{I(t)}{N}, \\ \dfrac{\mathrm{d}I(t)}{\mathrm{d}t} = \dfrac{I(t)}{N} \displaystyle\int_0^1 \beta(a) S(t,a)\, da - \gamma I(t), \\ \dfrac{\mathrm{d}R(t)}{\mathrm{d}t} = \gamma I(t), \end{cases} \tag{4}$$

The case $d = 0$ (heterogeneous but without variability) writes:

$$\begin{cases} \dfrac{\partial S(t,a)}{\partial t} = -\dfrac{\beta(a) S(t,a) I(t)}{N}, \\ \dfrac{\mathrm{d}I(t)}{\mathrm{d}t} = \dfrac{I(t)}{N} \displaystyle\int_0^1 \beta(a) S(t,a)\, da - \gamma I(t), \\ \dfrac{\mathrm{d}R(t)}{\mathrm{d}t} = \gamma I(t). \end{cases} \tag{5}$$

We list below some elementary mathematical properties of the model. First, we note that this model contains the traditional SIR model when the initial profile of susceptible $a \mapsto S_0(a)$ is uniform in a. Indeed, it is straightforward to see that then, $S(t,a)$ also does not depend on a.

The dynamics of total infectious individuals is governed by

$$\frac{dI(t)}{dt} = I(t) \left(\frac{1}{N} \int_0^1 \beta(a) S(t,a)\, da - \gamma \right), \tag{6}$$

so that the equivalent of the so called "effective R" coefficient in traditional SIR model, namely $\beta S(t)/(\gamma N)$, is replaced by

$$\frac{1}{N\gamma} \int_0^1 \beta(a) S(t,a)\, da. \tag{7}$$

Unlike traditional SIR model, in which the evolution of infectious $t \mapsto I(t)$ begins by an exponentially growing phase, has a unique maximum and tends to zero for large time, Model (4) may exhibit more sophisticated behaviours such as rebounds in I with multiple local maxima as well as plateaus. This property can be intuitively understood by analysing the evolution of the growth factor in (6):

$$\frac{1}{N} \frac{d}{dt} \int_0^1 \beta(a) S(t,a)\, da = -\frac{I(t)}{N^2} \int_0^1 \beta^2(a) S(t,a)\, da - \frac{d}{N} \int_0^1 \beta'(a) \frac{\partial S(t,a)}{\partial a}\, da. \tag{8}$$

In the absence of diffusion, $d = 0$, this growth factor is non increasing in time. For heterogeneous profiles and non zero diffusion, the first term of the right hand side is always negative whereas the second term may be positive if for instance $a \mapsto \beta(a)$ is increasing and $a \mapsto S(t,a)$ is a decreasing function. Below we show that it is always the case under some conditions. Thus, depending on the amplitude of the above two terms, the growth factor may have increasing and decreasing phases. It naturally leads one to consider initial conditions characterised by the property $S_0'(a)\beta'(a) < 0$ such as

$$\beta(a) = \beta_0 \exp\left(\frac{a^2}{\eta^2}\right) \quad \text{for some } \eta > 0, \tag{9}$$

$$S_0(a) = S_1 + (S_0 - S_1)\exp\left(-\frac{a^2}{\kappa^2}\right) \quad \text{for some } \kappa > 0 \text{ and } 0 < S_1 < S_0. \tag{10}$$

It means that individuals are sorted by increasing infection rate, according to the variable a, and that the vast majority of individuals have low infectiousness whereas few individual close to $a = 1$ have extremely risky behaviour. To some extent, social contact surveys [3–5] justify an initial distribution of susceptible individuals like (10). However, we are not aware of a rigorous justification of the expression (9) of the sharply increasing profile in β.

Let us now show that the evolution preserves the property of being decreasing in a for the profiles of the susceptible population.

Proposition 1. *If S_0 is a decreasing function of a, then $S(t,\cdot)$ is also decreasing in a for all time t.*
To prove this property, we simply note that the derivative $v(t,a) := \partial S(t,a)/\partial a$ of S with respect to a satisfies the following equation

$$\begin{cases} \dfrac{\partial v}{\partial t} + \beta \dfrac{I(t)}{N} v - d\dfrac{\partial^2 v}{\partial a^2} = -\beta'(a)S(t,a)\dfrac{I(t)}{N} \\ v(t,0) = v(t,1) = 0, \\ v(0,a) = \dfrac{\partial S(0,a)}{\partial a}. \end{cases} \tag{11}$$

Since the right hand side of the first equation of (11) is negative and the initial condition $v(0,a) \leq 0$, the parabolic maximum principle then shows that $v(t,a) \leq 0$ for all further time.

Further mathematical properties of Model (4)

Let us first notice that we can describe the dynamics of susceptible individuals in (4) in terms of its probability density $f(t,a) = S(t,a)/S(t)$ where $S(t) = \int_0^1 S(t,a)\,da$:

$$\frac{\partial f(t,a)}{\partial t} = -\frac{I(t)}{N} f(t,a)\left(\beta(a) - \bar{\beta}(t)\right) + d\frac{\partial^2 f(t,a)}{\partial a^2}, \tag{12}$$

where we denote $\bar{\psi}(t) = \int_0^1 \psi(t,a) f(t,a)\,da$. This equation shows the effect of infections on the distribution of susceptible population as a function of a. Indeed, it shows that, for instance in the absence of diffusion ($d = 0$), the proportion of the population of high a goes down as a result of infection affecting it more in relative terms than the remaining of the population. The presence of diffusion ($d > 0$) mitigates this effect by fuelling as it were the epidemic with individuals who had initial lower risk trait.

The equivalent of (8) for the average transmission rate $\bar{\beta}$ is

$$\frac{d\bar{\beta}(t)}{dt} = -\frac{I(t)}{N} Var(\beta)(t) - d\int_0^1 \beta'(a)\frac{\partial f(t,a)}{\partial a}\,da, \quad \text{where} \quad Var(\beta) = \overline{\beta^2} - \bar{\beta}^2. \tag{13}$$

In other words, in the absence of behavioural variability ($d = 0$), the average of the transmission rate decreases under the effect of its variance, the latter being directly linked to behavioural heterogeneity. Thus a higher heterogeneity promotes a faster decay of the average transmission rate. This is a remarkable property of the system in absence of diffusion or with small diffusion.

In the presence of diffusion $d > 0$, this effect is in competition with the second term of the right hand side of (13) which may be positive if for instance the profile $a \mapsto \beta(a)$ is increasing while the distribution f decreases along variable a.

Our next result concerns the effect of heterogeneity on herd immunity. We analyze it in the absence of diffusion.

Theorem 1. *Let $(t,a) \mapsto (S(t,a), I(t), R(t))$ be the solution of (5) (that is when $d = 0$) with initial conditions $S(0,a) = \tilde{S}_0 f_0(a)$, $I(0) = \tilde{I}_0 > 0$ and $R(0) = 0$ (for some positive constants $\tilde{S}_0$ and $\tilde{I}_0$). Then, $t \mapsto S(t,\cdot)$ tends as $t \to +\infty$ to a limit profile $a \mapsto S_\infty(a)$ in such a way that*

$$\int_0^1 S_\infty(a)\,da \geq \tilde{S}_\infty,$$

where $\tilde{S}_\infty > 0$ is the limit value as t goes to $+\infty$ of susceptible individuals $\tilde{S}(t)$ associated to the homogeneous SIR model (1) with initial conditions $(\tilde{S}_0, \tilde{I}_0, 0)$ and transmission rate $\bar{\beta}_0 = \int_0^1 \beta(a) f_0(a)\,da$.
Thus, this theorem asserts that heterogeneity lowers the herd immunity rate needed to stop an epidemic. Note that Several works study the asymptotic states of similar models without diffusion, in a discrete class framework. We mention in particular Magal et al.[6], Dolbeault and Turinici[7] and Almeida et al.[8].

Sketch of proof: The existence of the limit profile $a \mapsto S_\infty(a)$ follows from the monotonicity of $t \mapsto S(t,a)$ for fixed $a \in (0,1)$. Since $t \mapsto I(t)$ can be easily proved to vanish for large time, the recovered compartment $R(t)$ also tends to a limit R_∞. Using R as a time variable leads to:

$$I(R) = \tilde{I}_0 - R - \int_0^1 S_0(a)\left(\exp\left(-\frac{\beta(a)R}{N\gamma}\right) - 1\right) da,$$

so that

$$S_\infty(a) = S_0(a)\exp\left(-\frac{\beta(a)R_\infty}{N\gamma}\right)$$

and

$$\tilde{I}_0 = R_\infty + \int_0^1 S_0(a)\left(\exp\left(-\frac{\beta(a)R_\infty}{N\gamma}\right) - 1\right) da. \tag{14}$$

Equation (14) expresses $\tilde{I}_0$ as an increasing function F_β of R_∞, which is therefore uniquely defined. Application of Jensen's convexity inequality then leads to

$$G\left(\int_0^1 \beta(a) f_0(a)\, da\right) \leq \int_0^1 G(\beta(a)) f_0(a)\, da, \quad \text{where} \quad G(Z) = \exp(-ZR_\infty/(\gamma N)). \tag{15}$$

We deduce that $F_\beta(x) \geq F_{\bar{\beta}_0}(x)$ for all $x > 0$, where $F_{\bar{\beta}_0}$ is associated with the homogeneous SIR model (1) with initial conditions $(\tilde{S}_0, \tilde{I}_0, 0)$ and homogeneous parameter $\bar{\beta}_0$, which allows to conclude.

An interesting property of System (5) is the conservation of the following entropy-like quantity

$$V(t) = \int_0^1 S(t,a)\, da + I(t) - \frac{\gamma N}{\beta_m}\int_0^1 \log S(t,a)\, da, \quad \text{where} \quad \beta_m = \int_0^1 \beta(a)\, da. \tag{16}$$

The property $V(t) = V(0)$ can be deduced from (5).

Next, we consider more specifically the role of diffusion $d > 0$. First, we note that in the presence of diffusion, the above entropy V^d based on solutions $(S^d(t,a), I^d(t), R^d(t))$ of (4) is non-decreasing in a similar manner as in the framework of viscous perturbations of scalar conservation laws:

$$\frac{dV^d(t)}{dt} = -\frac{\gamma N d}{\beta_m}\int_0^1 \frac{(\partial_a S^d(t,a))^2}{S^d(t,a)}\, da \leq 0. \tag{17}$$

The following result emphasises the differences between the case with diffusion ($d > 0$) and the case without ($d = 0$).

Proposition 2. *Assume $d > 0$ and consider solutions $(S^d(t,a), I^d(t), R^d(t))$ of (4). The following properties hold:*

(i) $t \mapsto S^d(t,a)$ *is not necessarily decreasing in time for $a \in (0,1)$ given.*

(ii) *If $\mathscr{R}_0 = \int_0^1 S_0^d(a)\beta(a)\, da/(N\gamma) > 1$, then there exists $t_0 > 0$ such that $t \mapsto I^d(t)$ is increasing for $t \in (0, t_0)$.*

(iii) *If $\mathscr{R}_0 < 1$, $t \mapsto I^d(t)$ is decreasing in time over a finite time interval. Still, there are situations where I^d can be increasing over some later time intervals.*

We will give the proof of this result in a forthcoming work.

We now compare the combined impact of heterogeneity and variability compared with the case of homogeneous populations.

Theorem 2. *For given $d > 0$, let $(S^d(t,a), I^d(t), R^d(t))$ be solution of (4) with initial conditions $(S_0^d(a) = S_0(a), I^d(0) = I_0, R^d(0) = 0)$. The following results holds*

(i) *$S^d(t,\cdot)$ converges as t goes to $+\infty$ to a positive constant S_∞^d independent of a.*

(ii) *Let $(\bar{S}(t), \bar{I}(t), \bar{R}(t))$ be solution of the homogeneous SIR model (1) with initial conditions $(\bar{S}(0) = \int_0^1 S_0(a)\, da, \bar{I}(0) = I_0, \bar{R}(0) = 0)$ and transmission rate $\beta_m = \int_0^1 \beta(a)\, da$. Then, denoting $\bar{S}_\infty$ the large time limit of $t \mapsto \bar{S}(t)$, one has $S_\infty^d > \bar{S}_\infty$.*

We already know from Theorem 1 above that heterogeneity is beneficial in terms of herd immunity. From this point of view, the effect of variability is a priori not so clear because the constant shuffling of population keeps fuelling as it were the epidemic. However, part (ii) in the previous result shows that heterogeneity and variability still have a positive effect on herd immunity (that is $S^d_\infty > \bar{S}_\infty$).

The convergence of solutions to states that do no depend on a is natural because of the role of diffusion. Indeed, the diffusion term takes over once the $I(t)$ term becomes small and thus the first equation describes a diffusion. But this only happens in the long term and the relevance here is rather at intermediate times.

Sketch of proof: Assuming that the initial profile S_0 and β are regular enough in the a variable, integration by parts of the S equation of (4) multiplied by $\partial_t S$ leads to:

$$\int_0^1 \frac{S^d(t,a)^2}{2} da + \int_0^t I^d(\tau) \int_0^1 \frac{\beta(a) S^d(\tau,a)^2}{N} d\tau da + \int_0^t \int_0^1 d\, |\partial_a S^d(\tau,a)|^2 \, d\tau da$$

$$= \int_0^1 \frac{S_0^d(a)^2}{2} da \quad \text{for all} \quad t \geq 0. \tag{18}$$

Moreover,

$$\int_0^t \int_0^1 |\partial_t S^d(\tau,a)|^2 \, d\tau da + \int_0^1 d\, \frac{|\partial_a S^d(t,a)|^2}{2} da = -\int_0^t \int_0^1 \frac{\beta(a) S^d(\tau,a) I^d(\tau)}{N} \partial_t S^d(\tau,a)\, d\tau da$$

$$+ \int_0^1 d\, \frac{|\partial_a S_0^d(a)|^2}{2} da. \tag{19}$$

Hence,

$$\int_0^t \int_0^1 |\partial_t S^d(\tau,a)|^2 \, d\tau da + \int_0^1 d\, \frac{|\partial_a S^d(t,a)|^2}{2} da \leq \frac{1}{2} \int_0^t \int_0^1 |\partial_t S^d(\tau,a)|^2 \, d\tau da$$

$$+ \sup_{\tau \geq 0} \frac{I^d(\tau)}{N} \sup_{a\in(0,1)} \beta(a) \int_0^t \int_0^1 \frac{\beta(a) S^d(\tau,a)^2 I^d(\tau)}{2N} d\tau da + \int_0^1 d\, \frac{|\partial_a S_0^d(a)|^2}{2} da \quad \text{for all} \quad t \geq 0, \tag{20}$$

so that we have

$$\int_0^t \int_0^1 |\partial_t S^d(\tau,a)|^2 \, d\tau da + \int_0^1 d\, |\partial_a S^d(t,a)|^2 \, da \leq \int_0^1 d\, |\partial_a S_0^d(a)|^2 \, da$$

$$+ \sup_{a\in(0,1)} \beta(a) \int_0^t \int_0^1 \frac{\beta(a) S^d(\tau,a)^2 I^d(\tau)}{N} d\tau da. \tag{21}$$

It follows that $\partial_a S^d(t,\cdot)$ converges to 0 in $L^2(0,1)$: if it does not hold, there exists $(t_n)_{n\in\mathbb{N}} \to +\infty$ and $\alpha > 0$ such that $\|\partial_a S(t_n,\cdot)\|^2_{L^2(0,1)} \geq \alpha$. On the other hand, there exists T such that

$$\sup_{a\in(0,1)} \beta(a) \int_T^{+\infty} \int_0^1 \frac{\beta(a) S^d(\tau,a)^2 I^d(\tau)}{N} d\tau da \leq \frac{\alpha}{2}.$$

From (21) generalised between two positive times, for all $t > T$, there exists n such that $t_n > t$ and

$$\int_0^1 d\, |\partial_a S^d(t_n,a)|^2 \, da \leq \int_0^1 d\, |\partial_a S^d(t,a)|^2 \, da + \frac{\alpha}{2}, \quad \text{so that} \quad \int_0^1 d\, |\partial_a S^d(t,a)|^2 \, da \geq \frac{\alpha}{2},$$

which contradicts the integrability of $\partial_a S$ in $L^2(\mathbb{R}^+ \times (0,1))$. Next, from (21), we deduce that $S^d(t,\cdot)$ is bounded in $H^1(0,1)$ uniformly in time, so that it is compact in $C^{0,1/2}(0,1)$: a sequence $(t_n)_{n\in\mathbb{N}} \to +\infty$ exists such that $S^d(t_n,\cdot)$ converges in

$C^{0,1/2}(0,1)$ to some $S^d_\infty \in H^1(0,1)$ as n goes to $+\infty$. Since $\partial_a S^d_\infty = 0$, S^d_∞ is a constant. In order to prove the uniqueness of S^d_∞, we observe that for all $(t,t') \in \mathbb{R}^{+2}$

$$\frac{1}{2}\int_0^1 \left(S^d(t,a)^2 - S^d(t',a)^2\right) da = -d\int_{t'}^{t}\int_0^1 |\partial_a S^d(\tau,a)|^2 \, d\tau da - \int_{t'}^{t}\int_0^1 \frac{\beta(a) I^d(\tau) S^d(\tau,a)}{N} \, d\tau da, \tag{22}$$

and use the fact that $\partial_a S \in L^2(\mathbb{R}^+ \times (0,1))$, $S/N \leq 1$, and $I \in L^1(\mathbb{R}^+)$.

In order to prove (ii), using the fact that $dR^d(t)/dt = \gamma I^d(t) \geq$ and $R^d(t) \leq N$, we deduce that $R^d(t)$ converges to some $R^d_\infty > 0$. The conservation of total population $\int_0^1 S^d(t,a)\,da + I^d(t) + R^d(t) = 1$ then yields the convergence of $I^d(t)$ to 0 as $t \to +\infty$.

Integrating (17) between 0 and t, and letting t go to $+\infty$ leads to

$$S^d_\infty - \frac{\gamma N}{\beta_m} \log S^d_\infty = V^d(0) - d\int_0^{+\infty}\int_0^1 \frac{|\partial_a S^d(t,a)|^2}{S^d(t,a)} dt da, \tag{23}$$

which yields the estimate $S^d_\infty > \bar{S}_\infty$. The detailed proofs and further developments are postponed to a forthcoming work.

Some simulations with rebounds and plateaus

Some simulations show that the model adequately captures dynamical features such as epidemiological rebounds and plateaus. The initial conditions considered here are (10) where $\kappa = 0.44$, $S_0 = 90$ and $S_1 = 119,000$ (total initial susceptible individuals are around $46,398$), initial infected people $I(0) = 1$, and β is given by (9) for $\beta_0 = 0.008$ and η is taken such that $\beta(1) = 26$.

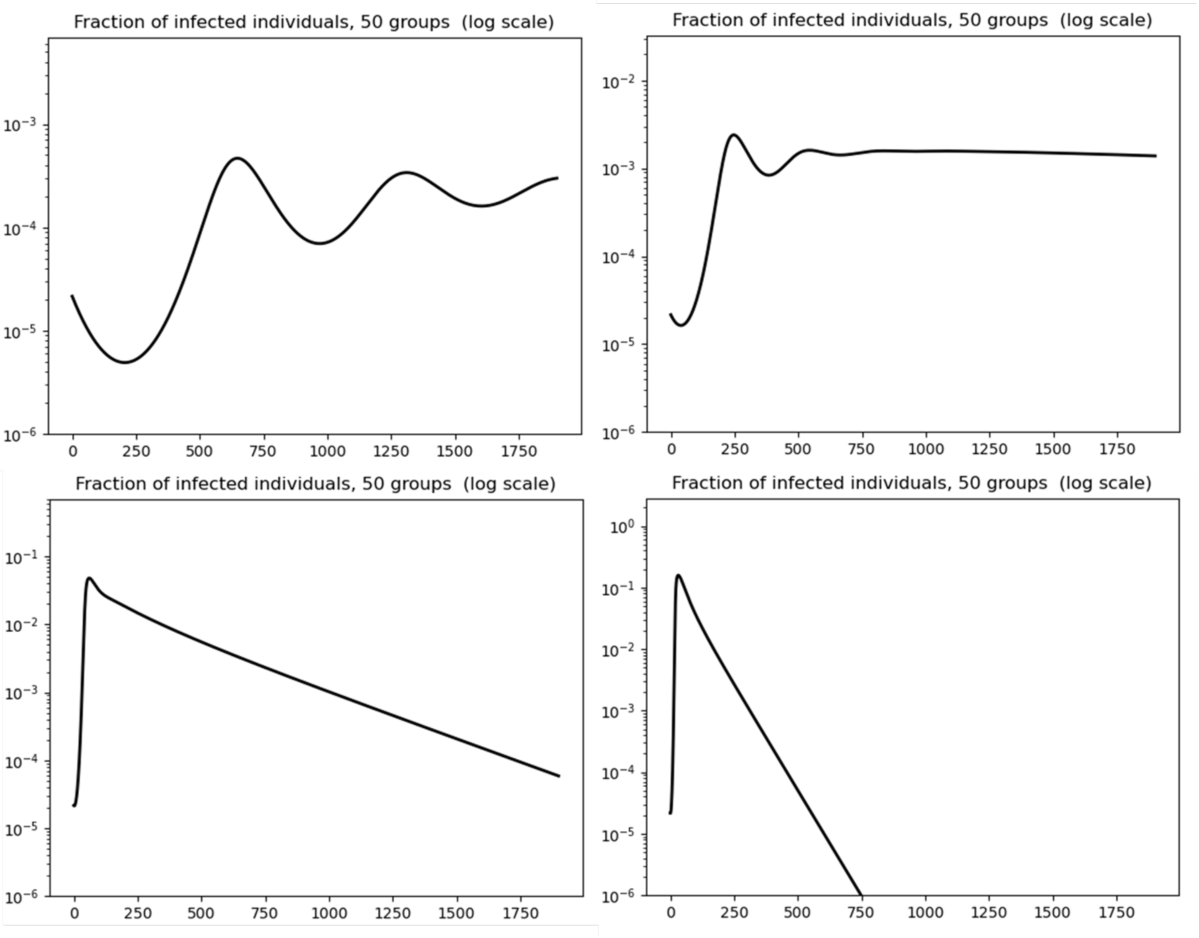

Figure S9. Model behaviour depending on diffusion parameter values: infected rate dynamics in logarithmic scale. From left to right and then top to bottom, graphs are associated with $d = 10^{-5}$, $d = 5 \cdot 10^{-5}$, $d = 10^{-3}$ and $d = 5 \cdot 10^{-3}$ (in day^{-1} unit)

Diffusion of susceptible individuals seem to be the most sensitive parameter in order to obtain either traditional SIR type behaviour or rebounds and plateau-type dynamics. Figure S9 illustrates the changes of dynamics depending on diffusion

parameter d. The associated dynamics of susceptible individuals are represented in Figure S10 when variable $a \in (0,1)$ is discretized into $n_g = 50$ groups through a finite difference scheme. Coloured curves represent each discretized group and the black curve the average of the groups (total susceptible individuals).

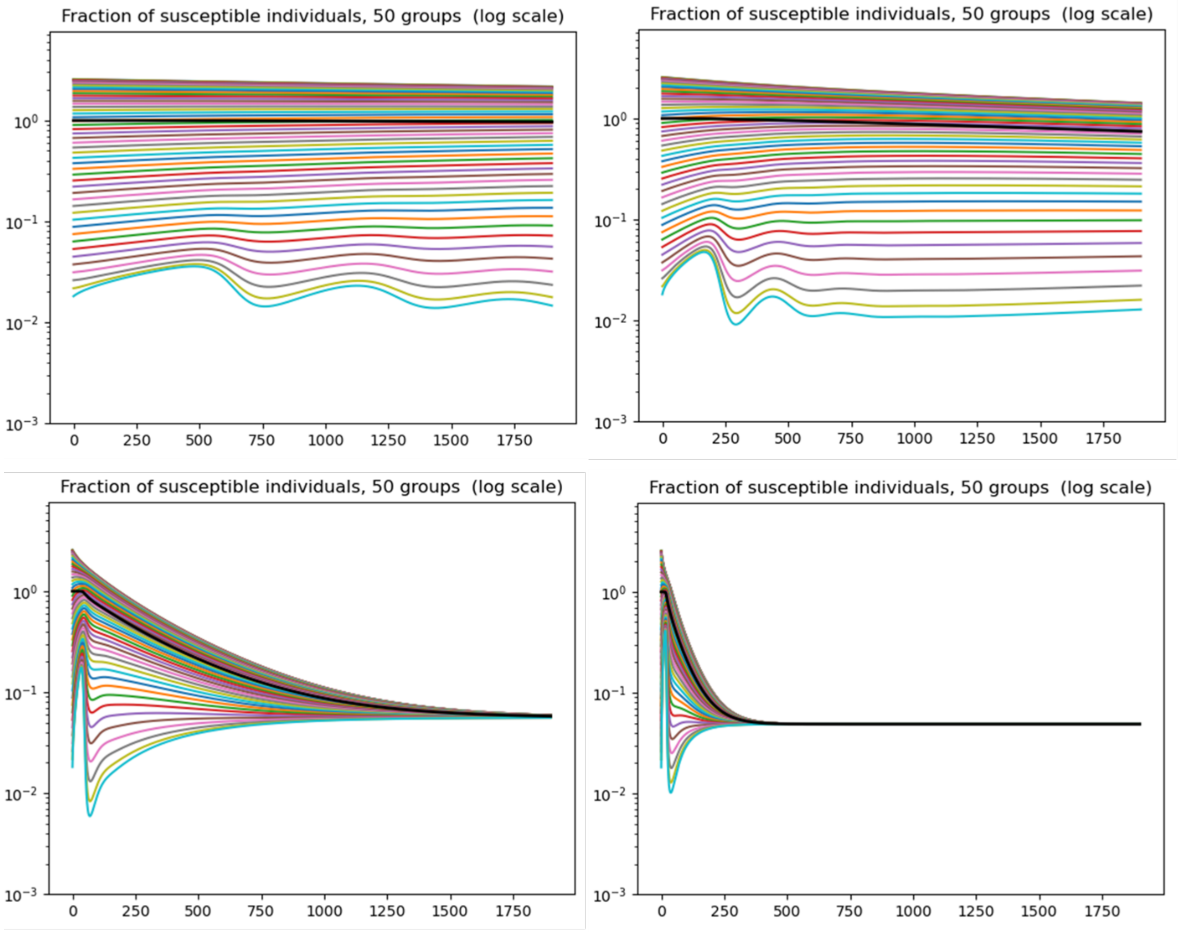

Figure S10. Model behaviour depending on diffusion parameter values: 50 groups of susceptible individuals, in logarithmic scale. The black curve corresponds to total susceptible individuals and the bottom light blue curve is associated with the most risky behaviour (a close to 1) and may not be monotonically decreasing in time. From left to right and then top to bottom, graphs are associated with $d = 10^{-5}$, $d = 5 \cdot 10^{-5}$, $d = 10^{-3}$ and $d = 5 \cdot 10^{-3}$ (in day^{-1} unit)

One can observe the effect of diffusion: while individuals with the riskiest behaviour are rapidly consumed, their group is renewed through diffusion from the majority of less infectious individuals. Depending on the parameter range of the diffusion coefficient d in (4), it leads to multiple epidemic waves, plateaus, or classical single wave SIR-like dynamics.

Stability of plateaus: the key role of diffusion

Let us illustrate in a heuristic manner the impact of diffusion on the existence and stability of plateaus in the dynamics of infectious individuals in Model (4). First, the second derivative of $x = \log I$ satisfies

$$\frac{d^2x}{dt^2}(t) = -I(t)\int_0^1 \frac{\beta^2(a)S(t,a)}{N^2}\,da - d\int_0^1 \beta'(a)\frac{\partial S(t,a)}{\partial a}\,da. \tag{24}$$

In the case $d = 0$, we deduce from (24) that $d^2x/dt^2(t) < 0$ for all $t \in \mathbb{R}^+$, so that $x = \log I$ is a strictly concave function of time. The consequence is that only slow decrease at the beginning of the decay phase can be obtained. Plateaus or shoulder-like patterns cannot arise in the case $d = 0$, nor rebounds since $x(t)$ has a single maximum after which it keeps decreasing.

In the case $d \neq 0$, Equation (24) can be reformulated as

$$\frac{d^2x}{dt^2}(t) = \eta(t)\left(1 - \frac{I(t)}{I_p(t)}\right), \tag{25}$$

where

$$\eta(t) = -\frac{d}{N}\int_0^1 \beta'(a)\frac{\partial S(t,a)}{\partial a}\,da \quad \text{and} \quad I_p(t) = \frac{N^2\eta(t)}{\displaystyle\int_0^1 \beta^2(a)S(t,a)\,da}. \tag{26}$$

Assuming that β and S_0 are respectively increasing and decreasing functions of a, we deduce from Proposition 1 that $\eta(t)$ and $I_p(t)$ are always positive. Introducing $x_p(t) = \log I_p(t)$, Equation (25) can be rewritten as

$$\frac{d^2x}{dt^2}(t) + \eta(t)\left(\exp[x(t) - x_p(t)] - 1\right) = 0. \tag{27}$$

It means that $d^2x/dt^2(t)$ may be positive (resp. negative) depending on whether $x(t)$ is lower (resp. greater) than $x_p(t)$. In particular, in the neighborhood of times t such that $x(t)$ is close to $x_p(t)$,

$$\exp[x(t) - x_p(t)] - 1 \approx x(t) - x_p(t),$$

so that oscillations may arise, leading to patterns such as the ones seen on Figure S9. These oscillations also explain rebounds, or shoulders-like patterns before plateaus. Of course, this is not a proof but an indication to this effect and it warrants further mathematical investigation.

More general systems

First, it should be noted that the coefficient $\beta(a)$ can be time dependent: $\beta = \beta(t,a)$. We can thus incorporate public health policies changes such as social distancing, lockdowns etc. For instance, we can consider a transition between two profiles $\beta_1(a)$ and $\beta_2(a)$ at a given date.

Then, we observe that System (4) is a particular case of a more general class of models. Indeed, we may want to also consider heterogeneity in the compartment of infected. This for instance reflects the division between symptomatic and asymptomatic individuals, or other differences. In particular, in terms of behaviour one can then include effects such as "super-spreaders". We then get a model where the population of infected also depends on the same parameter a, that is $I = I(t,a)$. We assume that the probability of interaction between a susceptible of class a and an infected of class b is given by a coefficient of transmission $\mathfrak{B}(a,b)$. These considerations lead us to the following general system.

$$\begin{cases} \dfrac{\partial S(t,a)}{\partial t} - d\,\dfrac{\partial^2 S(t,a)}{\partial a^2} = -\dfrac{S(t,a)}{N}\displaystyle\int_0^1 \mathfrak{B}(a,b)I(t,b)\,db, \\ \dfrac{\partial I(t,a)}{\partial t} = \dfrac{S(t,a)}{N}\displaystyle\int_0^1 \mathfrak{B}(a,b)I(t,b)\,db - \gamma I(t,a), \\ \dfrac{\mathrm{d}R(t)}{\mathrm{d}t} = \gamma\displaystyle\int_0^1 I(t,a)\,da. \end{cases} \tag{28}$$

We observe that our model above (4) is derived from this more general class by assuming that $\mathfrak{B}(a,b)$ is independent of b. In fact, the same is true for a finite number of states. This type of modelling of heterogeneity can be found in Magal et al.[6] and Arino et al.[9] in the discrete setting (a has a finite number of values) and without diffusion ($d = 0$). These works do not seem to yield shoulder, plateau and rebound-type patterns.

For simplicity we assume that the decay rate γ is uniform. Note that the second equation states that new infected individuals of class a result from an infection of a susceptible individual of the same class. Other choices are possible, but then, in order to be consistent, one would need to introduce new compartments such as symptomatic/asymptomatic, hospitalised etc. Likewise, we could consider other variants of the SIR model, such as the SEIR model. It would then be natural to assume that exposed individuals also move around. We are then led to a system of the following kind.

$$\begin{cases} \dfrac{\partial S(t,a)}{\partial t} - d\,\dfrac{\partial^2 S(t,a)}{\partial a^2} = -\dfrac{S(t,a)}{N}\displaystyle\int_0^1 \mathfrak{B}(a,b)I(t,b)\,db, \\ \dfrac{\partial E(t,a)}{\partial t} - d\,\dfrac{\partial^2 E(t,a)}{\partial a^2} = \dfrac{S(t,a)}{N}\displaystyle\int_0^1 \mathfrak{B}(a,b)I(t,b)\,db - \rho E, \\ \dfrac{\partial I(t,a)}{\partial t} = \rho E - \gamma I(t,a), \\ \dfrac{\mathrm{d}R(t)}{\mathrm{d}t} = \gamma\displaystyle\int_0^1 I(t,a)\,da. \end{cases} \tag{29}$$

We can also consider a more general diffusion process on the behavioural variables than Brownian diffusion. We already mentioned above (3) the version involving a diffusion $\partial^2_{aa}[\sigma^2(a)S(t,a)]$ rather than $d\,\partial^2_{aa}S(t,a)$.

With a somewhat different point of view, we can assume that there is a probability kernel $K(a,b)$ that represents the probability distribution that an individual with trait b jumps to behaviour a. Then, the variation of S involves an integral operator rather than a heat operator. We are then led to the following system.

$$\begin{cases} \dfrac{\partial S(t,a)}{\partial t} = \displaystyle\int_0^1 K(a,b)S(t,b)\,db - S(t,a) - \beta(a)S(t,a)\frac{I(t)}{N}, \\ \dfrac{\mathrm{d}I(t)}{\mathrm{d}t} = \dfrac{I(t)}{N}\displaystyle\int_0^1 \beta(a)S(t,a)\,da - \gamma I(t), \\ \dfrac{\mathrm{d}R(t)}{\mathrm{d}t} = \gamma I(t), \end{cases} \tag{30}$$

System (30) is supplemented with the same type of initial conditions as above (4). Note that there are no boundary conditions in this case.

It is classical that one can recover System (4) as a certain limit of the more general class of systems (30) (compare e.g. the article by the first author et al.[10]). However, here it is interesting to look at more behavioural assumptions on the kernel K. For instance, it is natural to assume that $K(a,b)$ is singular when b is close to a, that $K(a,b)$ is rather large when b is close to 1, whereas it is very small when b is small. We leave the study of such kernels for future study.

Digital PCR in the framework of wastewater based epidemiology

Wastewater-based surveillance has been used over the past two decades to assess in real time the exposure of people to chemicals (in particular pollutants, licit and illicit or misused therapeutic or other drugs) and pathogens (bacteria or viruses) at the community level[11]. It provides a global picture of population health at the scale of an urban area connected to wastewater treatment plants (WWTP). Fully complying with the privacy of individuals, this approach rests on an explicit, objective observation. It also has the advantage to serve as an early warning system. Nonetheless, there are also uncertainties in the quantitative interpretation of these measurements. In particular, dilution due to storm-water, infiltration of parasite inflow water, varying population, bio-marker degradation, varying time spent in the flow..., all generate a variety of biases.

In spite of these uncertainties, there is a renewed interest in wastewater based epidemiology (WBE), in the context of the Covid-19 outbreak. There are numerous initiatives over the world, in Australia[12], Japan[13], Netherlands[14], Detroit[15], Paris[16], Spain[17] and Canada[18]. The recent periodic sampling campaigns in WWTPs rely on classical quantitative real time PCR (qRT-PCR) to estimate the concentration of SARS-Cov-2. At this stage, these programs serve as early warning platforms based on the observation that they detect SARS-Cov-2 in sewers at least seven days ahead of individual testings[15,17,19].

Only a few of them also used digital PCR on sub-samples to assess the measurement uncertainties of qRT-PCR measurements. Even if the benefit of dPCR in particular for low viral loads is mentioned[18], it is not currently used routinely for systematic measurements, mainly for economic reasons.

Plateau-type patterns at country level

In most European countries, Covid-19 data exhibit similar plateau-type features, as illustrated in the so called effective R coefficient computed from the daily number of deaths[20]. This effective R coefficient exhibits phases where it stabilises around 1, which corresponds to an epidemic plateau.

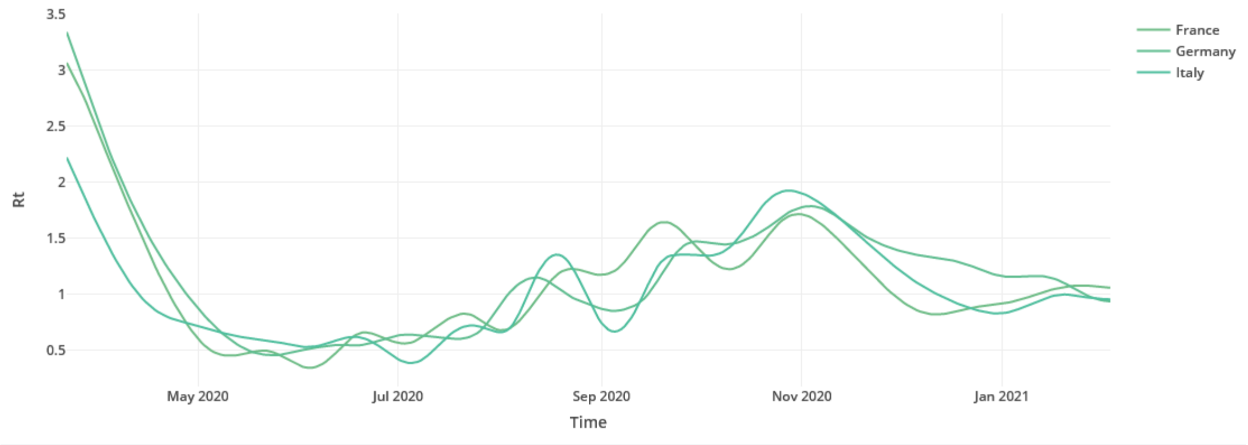

Figure S11. Effective R coefficient as a function of time[21] https://shiny.biosp.inrae.fr/app_direct/mapCovid19/.

Campo et al.[22] study more specifically the dynamics of Covid-19 in Italy and put emphasis on plateaus and multi-wave patterns, for which they discuss meta-stability properties.

Press articles illustrate the political leaders' observation that the Covid-19 outbreak has reached a plateau phase: Reuters, January 21st, 2021[23]; French government information service, December 10th, 2020[24]; A. Merkel, Westdeutsche Zeitung, January 21st, 2021[25]; E. Macron, February 2nd, 2021, interview on TF1 TV channel[26]).

References

1. Bauer, F., van den Driessche, P. & Wu, J. Mathematical epidemiology. *Lect. Notes Math.* **1945** (2008).
2. Biscani, F. & Izzo, D. A parallel global multiobjective framework for optimization: pagmo. *J. Open Source Softw.* **5**, 2338, DOI: https://doi.org/10.21105/joss.02338 (2020).
3. Stroud, P., del Valle, S., Sydoriak, S., Riese, J. & Minszewski, S. Spatial dynamics of pandemic influenza in a massive artificial society. *J. Artif. Soc. Soc. Simul.* **10** (2007).
4. Ibuka, Y. *et al.* Social contacts, vaccination decisions and influenza in japan. *J. Epidemiol. Community Heal.* **70**, 152–167, DOI: http://dx.doi.org/10.1136/jech-2015-205777 (2016).
5. Chan, H., Skali, A., Savage, D., Stadelmann, D. & Torgler, B. Risk attitudes and human mobility during the covid-19 pandemic. *Nat. scientific reports* **10**, DOI: https://doi.org/10.1038/s41598-020-76763-2 (2020).
6. Magal, P., Seydi, O. & Webb, G. Final size of a multigroup sir epidemic model: Irreducible and non-irreducible modes of transmission. *Math. Biosci.* **301**, 59–67, DOI: https://doi.org/10.1016/j.mbs.2018.03.020 (2018).
7. Dolbeault, J. & Turinici, G. Social heterogeneity and the Covid-19 lockdown in a multi-group SEIR model. *MedRxiv* **1**, DOI: https://doi.org/10.1101/2020.05.15.20103010 (2021).
8. Almeida, L., Bliman, P., Nadin, G., Perthame, B. & Vauchelet, N. Final size and convergence rate for an epidemic in heterogeneous population. *arXiv* **1** (2020).
9. Arino, J., Davis, J., Hartley, D. & Jordan, R. A multi-species epidemic model with spatial dynamics. *Math. Medicine Biol.* **22**, 129–142, DOI: https://doi.org/10.1093/imammb/dqi003 (2005).
10. Berestycki, H., Roquejoffre, J.-M. & Rossi, L. The periodic patch model for population dynamics with fractional diffusion. *Discret. Continuous Dyn. Syst. - series S* **4**, 1–13, DOI: http://dx.doi.org/10.3934/dcdss.2011.4.1 (2011).
11. Lorenzo, M. & Picó, Y. Wastewater-based epidemiology: current status and future prospects. *Curr. Opin. Environ. Sci. & Heal.* **9**, 77–84, DOI: https://doi.org/10.1016/j.coesh.2019.05.007 (2019).
12. Ahmed, W. *et al.* First confirmed detection of sars-cov-2 in untreated wastewater in australia: a proof of concept for the wastewater surveillance of covid-19 in the community. *Sci. Total. Environ.* **728**, DOI: https://doi.org/10.1016/j.scitotenv.2020.138764 (2020).
13. Haramoto, E. *et al.* First environmental surveillance for the presence of SARS-CoV-2 RNA in wastewater and river water in Japan. *Sci. Total. Environ.* **737**, DOI: https://doi.org/10.1016/j.scitotenv.2020.140405 (2020).

14. Medema, G. *et al.* Presence of SARS-Coronavirus-2 RNA in sewage and correlation with reported covid-19 prevalence in the early stage of the epidemic in the Netherlands. *Environ. Sci. Technol. Lett.* **7**, 511–516, DOI: https://dx.doi.org/10.1021/acs.estlett.0c00357 (2020).

15. Miyani, B. *et al.* SARS-Cov-2 in Detroit wastewater. *J. Environ. Eng.* **146**, DOI: https://doi.org/10.1061/(ASCE)EE.1943-7870.0001830 (2020).

16. Wurtzer, S. *et al.* Evaluation of lockdown impact on SARS-Cov-2 dynamics through viral genome quantification in paris wastewater. *www.eurosurveillance.org* **1**, DOI: https://doi.org/10.1101/2020.04.12.20062679 (2020).

17. Randazzo, W. *et al.* SARS-CoV-2 RNA titers in wastewater anticipated covid-19 occurrence in a low prevalence area. *Water Res.* **181**, DOI: https://dx.doi.org/10.1016%2Fj.watres.2020.115942 (2020).

18. d'Aoûst, P. *et al.* Quantitative analysis of SARS-Cov-2 RNA from wastewater solids in communities with low covid-19 incidence and prevalence. *Water Res.* **188**, DOI: https://doi.org/10.1101/2020.08.11.20173062 (2021).

19. Peccia, J. *et al.* Measurement of SARS-CoV-2 RNA in wastewater tracks community infection dynamics. *Nat. biotechnology* **38**, 1164–1167, DOI: https://doi.org/10.1038/s41587-020-0684-z (2020).

20. Roques, L., Bonnefon, O., Baudrot, V., Soubeyrand, S. & Berestycki, H. A parsimonious approach for spatial transmission and heterogeneity in the covid-19 propagation. *Royal Soc. Open Sci.* **12**, DOI: https://doi.org/10.1098/rsos.201382 (2020).

21. Roques, L., Klein, E., Papaïx, J., Sar, A. & Soubeyrand, S. Using early data to estimate the actual infection fatality ratio from covid-19 in france. *Biology* **9**, DOI: https://doi.org/10.3390/biology9050097 (2020).

22. Campi, G. *et al.* Metastable states in plateaus and multi-wave epidemic dynamics of Covid-19 spreading in Italy. *arxiv* **1**, DOI: https://arxiv.org/abs/2102.03836 (2021).

23. Reuters. *"Fauci says U.S. coronavirus infections may be plateauing, vaccines should protect against new variants"*. Press release 2021-01-21 (2021). https://www.reuters.com/article/us-usa-biden-fauci-idUSKBN29Q2XH.

24. SIG France, Press conference by Prime Minister J. Casteix. *"Mais, je vous l'ai dit, cette amélioration marque le pas depuis une semaine. Nous sommes sur une sorte de plateau."* . Press release 2020-12-10 (2020). https://www.gouvernement.fr/partage/11951-conference-de-presse-du-premier-ministre-point-de-situation-sur-la-lutte-contre-la-covid-19.

25. Westdeutsche Zeitung. *"Mit Hilfe der geltenden einschneidenden Maßnahmen sei es zwar geschafft worden, die Zahlen auf einem gewissen Plateau zu halten."*. Press release 2021-01-15 (2021). https://www.wz.de/nrw/bund-laender-treffen-zu-schaerferen-corona-massnahmen-fuer-dienstag-angesetzt_aid-55699681.

26. Le Progrès, Interview of E. Macron. *"Aujourd'hui, nous sommes sur un plateau."*. Press release 2021-02-02 (2021). https://www.leprogres.fr/sante/2021/02/02/coronavirus-la-france-doit-valider-ce-mardi-le-vaccin-astrazeneca.
